# Pyrenebutyrate Pt(IV) Complexes with Nanomolar Anticancer Activity

**DOI:** 10.3390/pharmaceutics15092310

**Published:** 2023-09-13

**Authors:** Anife Ahmedova, Rositsa Mihaylova, Silviya Stoykova, Veronika Mihaylova, Nikola Burdzhiev, Viktoria Elincheva, Georgi Momekov, Denitsa Momekova

**Affiliations:** 1 Faculty of Chemistry and Pharmacy, Sofia University, 1, J. Bourchier Blvd., 1164 Sofia, Bulgaria; sstoykova@chem.uni-sofia.bg (S.S.); ahvm@chem.uni-sofia.bg (V.M.); ohnb@chem.uni-sofia.bg (N.B.); 2 Faculty of Pharmacy, Medical University-Sofia, 2 Dunav Street, 1000 Sofia, Bulgaria; rmihaylova@pharmfac.mu-sofia.bg (R.M.); gmomekov@pharmfac.mu-sofia.bg (G.M.); dmomekova@pharmfac.mu-sofia.bg (D.M.)

**Keywords:** platinum(IV) prodrugs, anticancer agents, cytotoxicity, cellular uptake, activation by reduction

## Abstract

Research on platinum-based anticancer drugs continuously strives to develop new non-classical platinum complexes. Pt(IV) prodrugs are the most promising, and their activation-by-reduction mechanism of action is being explored as a prospect for higher selectivity and efficiency. Herein, we present the anticancer potency and chemical reactivity of Pt(IV) complexes formed by linking pyrene butyric acid with cisplatin. The results from cytotoxicity screening on 10 types of cancer cell lines and non-malignant cells (HEK-293) indicated IC_50_ values as low as 50–70 nM for the monosubstituted Pt(IV) complex against leukemia cell lines (HL-60 and SKW3) and a cisplatin-resistant derivative (HL-60/CDDP). Interestingly, the bis-substituted complex is virtually non-toxic to both healthy and cancerous cells of adherent types. Nevertheless, it shows high cytotoxicity against multidrug-resistant derivatives HL-60/CDDP and HL-60/Dox. The reactivity of the complexes with biological reductants was monitored by the NMR method. Furthermore, the platinum uptake by the treated cells was examined on two types of cellular cultures: adherent and suspension growing, and proteome profiling was conducted to track expression changes of key apoptosis-related proteins in HL-60 cells. The general conclusion points to a possible cytoskeletal entrapment of the bulkier bis-pyrene complex that could be limiting its cytotoxicity to adherent cells, both cancerous and healthy ones.

## 1. Introduction

Platinum-based drugs have a long clinical history in cancer treatment, despite the known limitations of cisplatin—the first metal complex approved as an anticancer drug. Since then, chemists, together with medical and pharmaceutical professionals, elaborate the chemical inertness of metal complexes to achieve more selective and effective anticancer drugs [1]. Among the thoroughly studied non-classical platinum drugs are the Pt(IV) complexes that are regarded as “prodrugs”, since their cytotoxicity is exerted only after reduction to the cytotoxic Pt(II) parent complex [2]. The activation-by-reduction mechanism has continuously been explored for Pt(IV) anticancer prodrugs as a plausible way to limit their off-target reactivity and thereby the undesired side effects known for most of the FDA-approved Pt(II) drugs. Selective reduction inside the cancerous cells would render the Pt(IV) prodrugs with a superior selectivity profile [3]. Indeed, the first metal-based anticancer drug for oral application is the Pt(IV) complex satraplatin that entered clinical trials due to its promising therapeutic profile [4]. Although some clinical studies have been abandoned at stage III [5], research towards improvement of the therapeutic and reactivity profiles of Pt(IV) complexes is a continuing endeavor for many research groups [6,7,8,9,10,11]. Well-established synthetic protocols to obtain predesigned structures with suitably selected axial and equatorial ligands is a serious achievement in these research directions [12,13]. Common logic is to decorate the Pt(IV) analogs of approved Pt(II) anticancer drugs (cisplatin, carboplatin, and oxaliplatin) with biologically active ligands in the axial position and thus enhance the selectivity and therapeutic profiles of the formed Pt(IV) complexes through the liberation of at least two bioactive components as a result of reduction [14]. Such design approaches of multitargeted Pt(IV) complexes have exploited known FDA-approved drugs, including histone deacetylase (HDAC) inhibitors [10,15,16], non-steroidal anti-inflammatory drugs (NSAIDs) [17,18,19,20], and other biologically active molecules [11,21]. Despite the reported promising results, it still remains questionable whether multitargeting Pt(IV) complexes can outperform some well-established therapeutic regimens of concomitant treatment with several drugs in flexible combination protocols [21,22].

High motivation to further explore the therapeutic potential of Pt(IV) complexes stems from the fact that there are already numerous examples of highly cytotoxic Pt(IV) complexes with minimal growth-inhibiting concentrations (IC_50_) in the nanomolar range. Nanomolar cytotoxicity has been reported for Pt(IV) prodrugs bearing biologically active axial ligands such as cyclooxygenase inhibitors, also known as NSAIDs [17,18,19], as well as other enzyme inhibitors [16,23,24]. Through the random replacement of bioactive axial ligands, either with long alkyl chain carboxylates [25,26] or bulkier aromatic carboxylates, the nanomolar cytotoxicity of some of the studied series of Pt(IV) prodrugs has been preserved or even improved [27,28]. In alternative approaches, carboxylates were introduced as equatorial ligands, and mechanistic studies sought out the correlation of the complexes’ lipophilicity and chemical reactivity with their nanomolar cytotoxicity [29,30,31]. In other cases, the axial ligand is an endogenous biomolecule, such as melatonin in Melatplatin [32], and yet, it exhibits nanomolar cytotoxicity. 

**Figure 1:**
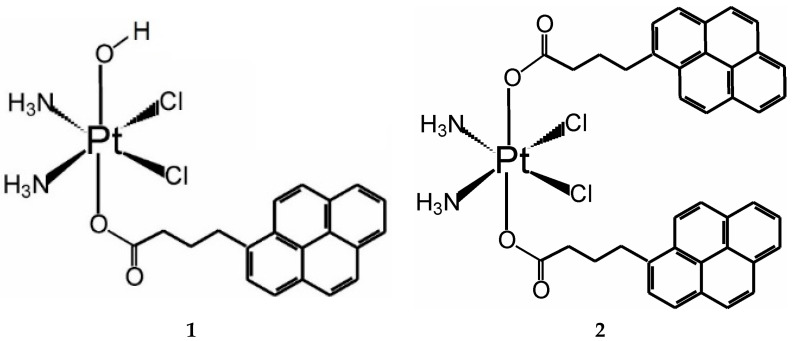
Structure of the mono- and disubstituted pyrenebutyrate complexes of Pt(IV), denoted as **1** and **2**, respectively.

Apparently, the combination of established guidelines and new breakthroughs is a promising approach in finding platinum-based drugs with improved anticancer activity. Therefore, we linked pyrene butyric acid with cisplatin and studied the chemical stability and anticancer profile of the formed Pt(IV) complexes. Pyrene came to our attention as a guest molecule that happens to modulate the anticancer activity of metallosupramolecular coordination capsules with remarkable selectivity to cancer cells [33]. As a strong intercalator, pyrene forms a rather robust host–guest complex with the cytotoxic supramolecular capsule, rendering it completely resistant to biological reductants and thereby practically non-toxic [34]. With the aim to employ this non-toxic host–guest complex as a delivery platform for anticancer drugs, we designed several potentially cytotoxic complexes that carried a pyrene butyric arm and could readily form host–guest complexes to be selectively delivered into cancer cells. For effective delivery of the cytotoxic part of this guest molecule, however, chemical detachment of the pyrene butyric arm should be achieved, as pyrene itself remains trapped in the host–guest complex. Therefore, Pt(IV) prodrugs with their known activation-by-reduction mechanism of action that proceed with the release of the axial ligands were the best candidates for this purpose. Herein, we report on the synthesis and structural characterization of two pyrenebutyric Pt(IV) complexes derived from cisplatin: compounds **1** and **2**, whose structures are depicted in Figure 1. Their cytotoxicity profile was evaluated in a panel of malignant and non-malignant human cell lines. NMR measurements were performed to study the reactivity of the complexes with biomolecules relevant to cancer evolution. Correlations were sought between the cytotoxicity of **1** and **2** and their chemical reactivity, as well as internalization in the cancer cells that was performed on two types of cells: adherent and suspension, for the sake of comparison. The latter was estimated by ICP-MS both in whole cells and in different cell fractions by using procedures from our previous studies on the mentioned metallosupramolecular capsules [35]. Moreover, a proteome analysis on HL-60 leukemia cells was performed in an attempt to identify the molecular pathways affected by the studied pyrenebutyrate Pt(IV) complexes.

## 2. Materials and Methods

### 2.1. Apparatus and Materials

N,N′-dicyclohexylcarbodiimide (DCC) and O-(benzotriazol-1-yl)-N,N,N′,N′-tetramethyluronium tetrafluoroborate (TBTU) were obtained from Acros Organics (Geel, Belgium). Additionally, 30% hydrogen peroxide, potassium tetrachlorido platinate(II), N-hydroxysuccinimide, and 1-pyrenebutyric acid were purchased from Sigma-Aldrich, Taufkirchen, Germany. A Nicolet 6700 Thermo Scientific FTIR spectrometer was used for recording the IR spectra in KBr, whereas a Bruker Avance III HD spectrometer (500.13 MHz for ^1^H and 125 MHz for ^13^C NMR) was used to obtain the NMR spectra. DMSO-*d*_6_ was used as the solvent, and its residual peaks were used for calibration of the chemical shifts that are provided in parts per million (δ). Data for the elemental analyses were obtained from a Vario EU III instrument. All solvents were of analytical or synthetic grade. For all in vitro experiments, cis-diamidodichlorido platinum(II) (CDDP), cisplatin, was provided by Sigma-Aldrich Chemie GmbH, Taufkirchen, Germany. A FractionPREP Cell Fractionation Kit from Bio Vision, Milpitas, CA USA, was used to perform the cell fractionation prior to the ICP-MS analyses. Buffer solutions were freshly prepared in deionized water.

### 2.2. Synthesis

Complex **1** was synthesized following the literature procedure for modification of the starting Pt(IV) analog of cisplatin-oxoplatin, *c,c,t*-[Pt(NH_3_)_2_Cl_2_(OH)_2_] (IR spectrum in Figure S1 in the Supplementary Materials), as described by Erxleben and coworkers [9]; namely, 0.8 eq. of the activated NHS ester of 1-pyrenebutyric acid (IR spectrum in Figure S2 in the Supplementary Materials) and oxoplatin (87.6 mg; 0.262 mmol) were stirred in DMSO at 50 °C for 24 h. Then, the solvent was evaporated, the residue was dissolved in 3 mL DMF, and the resulting complex was precipitated with diethyl ether (150 mL). The solid was isolated by centrifugation, washed several times with diethyl ether, left to airdry overnight, and stored under P_2_O_5_ (Yield, 91.9 mg; 0.152 mmol; 58% relative to Pt). Complex **2** was synthesized according to the procedure described by Gibson and coworkers [12] using TBTU as coupling agent and 1-pyrenebutyric acid in the presence of triethylamine, all in 3 equivalents to oxoplatin (58 mg, 0.174 mmol). After stirring the reaction mixture for 23 h at RT, the volume of the DMF solvent was reduced by evaporation, and the resulting complex was precipitated with acetone and filtered off, washed with acetone, air-dried, and stored under P_2_O_5_ (Yield, 107 mg; 70%). IR and NMR spectra (^1^H, ^13^C, ^195^Pt, and 2D) were used for structural characterization of the complexes and are provided in the Supplementary Materials, Figures S3–S26. 

Complex **1**: ^1^H NMR (DMSO-*d*_6_) δ: 1.97 (quint, 2H, H-3, J = 7.5 Hz); 2.34 (t, 2H, H-2, J = 7.1 Hz); 3.36 (t, 2H, H-4, J = 7.5 Hz); 5.80–6.20 (m, 6H, NH_3_); 7.98 (d, 1H, H-pyr, J = 7.8 Hz); 8.04 (t, 1H, H-pyr, J = 7.6 Hz); 8.10 (t, 1H, H-pyr, J = 8.9 Hz); 8.13 (d, 1H, H-pyr, J = 8.9 Hz); 8.21 (d, 2H, H-pyr, J = 8.8 Hz); 8.26 (t, 2H, H-pyr, J = 7.3 Hz); 8.47 (d, 1H, H-pyr, J = 9.3 Hz). ^13^C NMR (DMSO-*d*_6_) δ: 28.21 (1C, C-3); 32.33 (1C, C-2); 36.19 (1C, C-4); 124.04 (1C, C-pyr); 124.15 (1C, C-pyr); 124.20 (1C, C-pyr); 124.70 (1C, C-pyr); 124.81 (1C, C-pyr); 124.88 (1C, C-pyr); 126.09 (1C, C-pyr); 126.41 (1C, C-pyr); 127.17 (1C, C-pyr); 127.47 (1C, C-pyr); 127.77 (1C, C-pyr); 128.20 (1C, C-pyr); 129.21 (1C, C-pyr); 130.47 (1C, C-pyr); 130.89 (1C, C-pyr); 137.14 (1C, C-pyr); 180.78 (1C, C-1). ^195^Pt ^1^H NMR (DMSO-*d*_6_) δ: 1048 ppm. Calcd for Pt(NH_3_)_2_Cl_2_(C_20_H_15_O_2_)(OH) [C_20_H_22_Cl_2_N_2_O_3_Pt, MW = 604.38]: C 39.75%, H 3.67%, N 4.64%; found: C: 39.36%; H:3.39%; N: 4.52%; IR (ν, cm^−1^): 3481 m (ν_O-H_), 3234 s (ν_N-H_), 3202 s (ν_N-H_), 3143 s (ν_N-H_), 3040 s (ν_N-H_), 2960 m (ν_C-H_), 2870 m (ν_C-H_), 1644 vs. (ν_C=O_), 1620 s (ν_C=C_), 1596 s (ν_C-O_), 1558 m (ν_C-O_), 1435 w (ν_C-C_), 1401 m (ν_C-C_), 1369 m, 1356 m, 1287 m, 1254 m, 1097 w (δ_C-H_), 1046 w (δ_C-H_), 846 vs. (γ_C-H_). 

Complex **2**: ^1^H NMR (DMSO-*d*_6_) δ: 2.00 (quint, 4H, H-3, J = 7.5 Hz); 2.41 (t, 4H, H-2, J = 7.2 Hz); 3.38 (t, 4H, H-4, J = 7.5 Hz); 6.61 (br, s, 6H, NH_3_); 7.99 (d, 2H, H-pyr, J = 7.8 Hz); 8.05 (t, 2H, H-pyr, J = 7.6 Hz); 8.12 (d, 2H, H-pyr, J = 9.0 Hz); 8.14 (d, 2H, H-pyr, J = 9.0 Hz); 8.22 (d, 4H, H-pyr, J = 8.5 Hz); 8.26 (d, 2H, H-pyr, J = 7.5 Hz); 8.27 (d, 2H, H-pyr, J = 7.6 Hz); 8.47 (d, 2H, H-pyr, J = 9.3 Hz). ^13^C NMR (DMSO-*d*_6_) δ: 27.98 (2C, C-3); 32.13 (2C, C-2); 35.46 (2C, C-4); 123.96 (2C, C-pyr); 124.15 (2C, C-pyr); 124.22 (2C, C-pyr); 124.74 (2C, C-pyr); 124.86 (2C, C-pyr); 124.91 (2C, C-pyr); 126.12 (2C, C-pyr); 126.47 (2C, C-pyr); 127.22 (2C, C-pyr); 127.47 (2C, C-pyr); 127.79 (2C, C-pyr); 128.21 (2C, C-pyr); 129.26 (2C, C-pyr); 130.46 (2C, C-pyr); 130.90 (2C, C-pyr); 136.90 (2C, C-pyr); 180.63 (2C, C-1). ^195^Pt {^1^H}5NMR (DMSO-*d*_6_) δ: 1224 ppm. Calcd for Pt(NH_3_)_2_Cl_2_(C_20_H_15_O_2_)_2_ [C_40_H_36_Cl_2_N_2_O_4_Pt, MW = 874.71]: C 54.92%, H 4.15%, N 3.20%; found: C 55.30%; H 4.34%; N 3.40%. IR (ν, cm^−1^): 3246 s (ν_N-H_), 3185 s (ν_N-H_), 3039 m (ν_C-H_), 2939 m (ν_C-H_), 1686 s (ν_C=O_), 1654 s (ν_C=O_), 1603 s (ν_C=C_), 1416 w (ν_C-C_), 1361 s, 1340 s, 1318 s, 1281 m, 1221 m, 1204 m, 1182 w (δ_C-H_), 841 vs (γ_C-H_). 

### 2.3. Cell Lines and Culture Conditions 

In this work, we used ten types of human tumor cell lines, namely CASKI (cervical cancer); MDA-MB-231 (triple-negative breast cancer); MCF-7 (hormone-dependent breast cancer); HT-29 (colorectal carcinoma); T-24 (urinary bladder carcinoma); HL-60 (promyelocytic leukemia); HL-60/Dox (multi-resistant promyelocytic leukemia); HL-60/CDDP (CDDP-resistant promyelocytic leukemia); REH (precursor B-lymphoblastic leukemia); SKW-3 (acute T-lymphoblastic leukemia). The normal cell line of HEK-293 (human embryonic kidney cells) was used as a model for healthy cells to estimate the selectivity of the tested compounds. The HL-60/CDDP subline was developed through serial exposure of the HL-60 cell line to CDDP in increasing concentrations and thereafter sustained by cell cultivation in a growth medium with 25 μM CDDP. The multidrug-resistant (MDR) line HL-60/Dox was obtained from the German Cancer Research Center (DKFZ) in Heidelberg, Germany. Similarly, it was sustained by cell cultivation in the presence of 0.2 μM doxorubicin. All other cell lines were obtained from the German Collection of Microorganisms and Cell Cultures (DSMZ GmbH, Braunschweig, Germany) and were grown at 37 °C in an incubator BB 16-Function Line Heraeus (Kendro, Hanau, Germany) with a humidified atmosphere and 5% CO_2_. A growth medium of 90% RPMI-1640 + 10% FBS was used to maintain the cell lines. 

### 2.4. MTT Test for Cytotoxicity Assessment

The MTT method for assessing cell viability developed by Mosmann [36] was used to evaluate the in vitro cytotoxicity of the complexes. For this purpose, cells were harvested and seeded in 96-well plates at densities of 3 × 10^5^ for the suspension cultures and 1.5 × 10^5^ for the adherent ones. After 24 h of incubation, cells were treated with 2-fold serial dilutions of the compounds in the appropriate concentration range according to their chemosensitivity profiles. Treated cells were incubated for 72 h and were further processed according to the protocol described earlier [33,34,35]. A nonlinear regression analysis was applied for the estimation of the IC_50_ values from the concentration–response curves and using the curve fit (GraphPad Prizm 8.01 Software).

### 2.5. Proteome Analysis

A series of immunoassay experiments were performed to monitor changes in the proteome profile of cells treated with the newly synthesized metal complexes in a comparative manner to the referent drug cisplatin. Changes in the apoptotic and survival signaling of HL-60 cells in response to 24 h of exposure to CDDP and the studied Pt(IV) complexes **1** and **2** were tracked. Membrane-based sandwich immunoassays were conducted following the manufacturer’s protocol (Proteome Profiler Human Apoptosis Array Kit, R&D Systems Inc., Minneapolis, MN, USA). The arrays were visualized using a digital imaging system (Azure Biosystems C600, Dublin, CA, USA), and a densitometric analysis of the array spots was conducted using ImageJ^®^ 1.8.0 software. The most prominent changes in the proteome were expressed graphically relative to the untreated control and interpreted in a comparative manner to cisplatin as a reference compound.

### 2.6. ICP-MS Analysis

Determination of the platinum content in cells and cell fractions was performed by ICP-MS analysis on a Perkin Elmer SCIEX DRC-e inductively coupled plasma mass spectrometer equipped with a crossflow nebulizer. The mass spectrometer was optimized to ensure the maximum intensity of the Pt isotopes. The instrumental conditions for ICP-MS were described in [35]. The concentration of platinum in the analyzed samples was measured by the following isotopes: ^192^Pt, ^194^Pt, ^195^Pt, ^196^Pt, and ^198^Pt.

Platinum quantification was done by external calibration using standard solutions in the working range of 0.5–50 ppb and internal standard of 20 ppb ^191^Ir. All external standard solutions were prepared gravimetrically using fresh doubly deionized water (Millipore purification system Synergy, Molsheim, France). The correlation coefficient of >0.999 was estimated by the least-squares linear regression analysis of the measured platinum isotopes. 

The analyzed biological samples were free of Hf, as no interferences by hafnium oxides (^179^Hf^16^O and ^178^Hf^16^O) were detected on the platinum determination through its main isotopes: ^195^Pt and ^194^Pt by Q-ICP-MS [37]. The detection and quantification limits of the applied ICP-MS method were <0.004 ng/mL and <0.012 ng/mL, respectively.

All cell samples were allowed to stand for at least 48 h in 1 mL concentrated HNO_3_ (67–69%, Fisher Chemicals, Ultra Trace Metal Grade, Loughborough, UK) to ensure complete digestion and then diluted to 3 mL with water before being introduced into the plasma. Samples were analyzed as suspensions without filtration to avoid a loss in the platinum content. 

### 2.7. Total Protein Analysis

The cell lines (HT-29 and HL-60, 1 × 10^6^ cells/mL) were treated with compounds **1**, **2**, and CDDP at concentrations of 10 μmol/L and incubated for a period of 4 h. The medium was removed, and the cells were washed twice with cold PBS of pH 7.4. After centrifugation at 3200 rpm for 5 min, the cell pellets were resuspended in 100 μL 0.1% Triton X-100, and the samples were precipitated using an ice-cold bath for 45 min. The Smith method [38] was used for determination of the total protein content. Nontreated cells were processed as control samples.

### 2.8. Lactate Dehydrogenase (LDH) Activity Assay

The supernatants of the tumor cell lines (HT-29 and HL-60, 1 × 10^6^ cells/mL), treated with the test compounds at a concentration of 10 μmol/L for a period of 4 h, were used as test samples to assess the LDH activity. This was done using a commercial kit from Sentinel Diagnostics (Milano, Italy). A semiautomatic biochemical analyzer BA-88A (Shenzhen Mindray Bio-Medical Electronics, Shenzhen, China) was used for the measurements, and all steps were performed following the instructions of the manufacturer. Normal human serum DunaCont N (Diagnosticum Zrt., Budapest, Hungary) was used as the control for verification of the assay, while the supernatant from the untreated cells served as a blank sample.

### 2.9. Cell Fractionation for the ICP-MS Analysis

The HT-29 and HL-60 lines were seeded at a density of 1 × 10^6^ cells/mL for the platinum uptake study and treated with the tested compounds: **1**, **2**, and CDDP at a concentration of 10 μmol/L. After incubation for 4 h and removal of the medium, the cells were washed twice with PBS, centrifuged at 3200 rpm for 5 min, and the intact cells were collected for the estimation of the total platinum content. Simultaneously, a second set of samples was processed and subjected to cell fractionation according to the protocols provided by the manufacturer (FractionPREP Cell Fractionation Kit, Bio Vision). In consequence, four fractions were separated: the cytosolic, membrane/particulate, nuclear, and cytoskeletal fractions. The total cells and obtained cellular fractions were digested with 1 mL of 69% HNO_3_ for 48 h and subjected to ICP-MS measurements of the platinum content, as described in the previous section. 

## 3. Results

### 3.1. Synthesis and Stability of the Complexes

Complexes **1** and **2** were obtained after testing several different procedures for modification of the axial OH ligands of the Pt(IV) complexes [9,12,39]. For our case, coupling with 1-pyrenebutyric acid was achieved with the best yields in relatively mild and resource-efficient synthetic conditions using the following two methods: coupling with the NHS-activated ester of 1-pyrenebutyric acid, as described by Erxleben and coworkers [9], was used to obtain complex **1** with a 58% yield; complex **2** was obtained with a 70% yield using the coupling agent TBTU and following the procedure described by Gibson and coworkers [12]. In both cases, the reaction lasted no more than 24 h at mild (50 °C for **1**) or no heating (for **2**). The attachment of the pyrenebutyric fragment was unequivocally confirmed with ^1^H NMR and IR measurements by the presence of characteristic signals in the aromatic region (8–8.5 ppm) and the characteristic skeletal vibrations at ca. 850 cm^−1^, respectively. The signals for the methylene protons and their stretching vibrations were also clearly seen at the 2–3.4 ppm and at 2900–3040 cm^−1^ regions in the ^1^H NMR and IR spectra, respectively. The monosubstituted complex **1** was readily distinguished from the disubstituted complex **2** by the absence of the characteristic stretching vibration of the O-H bond at 3480 cm^−1^ in the IR spectrum of **2**. The integration of the ^1^H NMR signals also confirmed the attachment of two pyrenebutyric fragments to the oxoplatinum in complex **2**. This also affected the signals of the NH protons that are seen in the spectrum of **2** as a broad singlet at ca 6.6 ppm, whereas the ^1^H NMR spectrum of complex **1** reveals better-resolved ^1^*J*_NH_ and ^2^*J*_PtH_ couplings of near 50 Hz centered at ca. 6 ppm. Ultimately, the most definitive information to confirm the formation of mono- and bis-substituted complexes of Pt(IV) was obtained from their ^195^Pt NMR spectra that revealed clear signals at 1048 and 1224 ppm, respectively.

To test the stability of the complexes over time, we used NMR spectroscopy, as it is highly sensitive to eventual changes in the structures of the complexes. Both ^1^H and ^195^Pt NMR spectra were registered upon the addition of biological reductants, such as glutathione, glucose, or ascorbic acid, to DMSO solutions of complexes **1** and **2**. Qualitative changes were monitored at different time intervals after the addition of excess solid powder of the reductants. 

**Figure 2:**
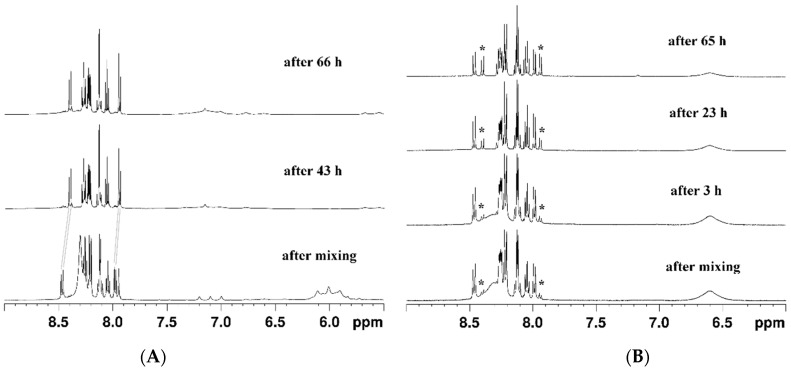
Changes in the ^1^H-NMR spectra upon the addition of ascorbic acid (in DMSO-*d*_6_): (**A**) of complex **1** over 66 h (grey lines denote the shift of the signals indicating the complete release of pyrenebutyrate) and (**B**) of complex **2** over 65 h (asterisks denote the appearance of the signals of free pyrenebutyrate).

**Figure 3:**
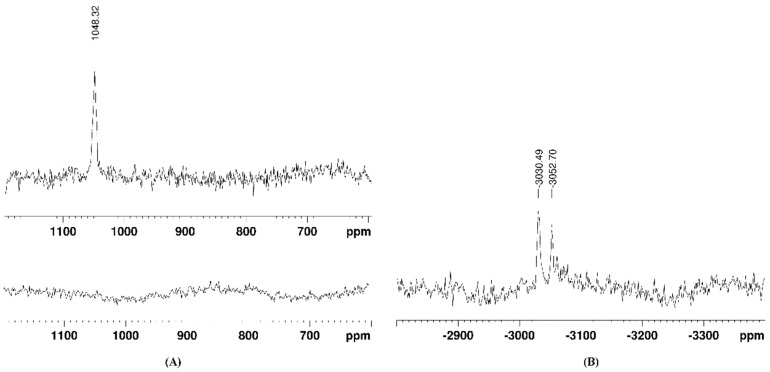
Changes in the ^195^Pt-NMR spectrum of complex **1** after 67 h of adding ascorbic acid (in DMSO-*d*_6_): (**A**) in the 400–1200 ppm region and (**B**) in the −2700 to −3500 ppm region.

Interestingly, the complexes were sensitive to the presence of ascorbic acid but remained intact upon the addition of glutathione or glucose (see the Supplementary Materials). A general observation from the NMR-monitored kinetics was that complex **1** was far more reactive towards ascorbic acid than complex **2**. This was evidenced by the changes observed in the ^1^H and ^195^Pt NMR spectra depicted in Figure 2 and Figure 3, respectively. 

Upon reaction with ascorbic acid, complex **1** rapidly released the pyrenebutyrate axial ligands, and the process was completed in two days. This was clearly seen from the changes in the aromatic region of the ^1^H NMR spectrum, highlighted with grey lines in Figure 2A. Similar changes started to evolve in the spectrum of complex **2** with added ascorbic acid, but it only partially converted to the reduced product, even after three days (Figure 2B).

The reduction of complexes **1** and **2** was confirmed by the loss of the characteristic NMR signal for Pt(IV) nuclei. By a thorough search in the broad ^195^Pt NMR spectral ranges (from 3000 to −4000 ppm), the signals of the formed Pt(II) species of the fully reduced complex **1** were detected at −3030 and −3052 ppm, as can be seen in Figure 3B. These signals could be attributed to Pt(II) complexes with DMSO, either S- or O-bound, as have earlier been reported by Sadler and coworkers [40]. Signals in the ^195^Pt NMR spectra of complex **2** could not be identified, most probably due to its partial reduction and the formation of various intermediate species in low quantities. 

### 3.2. Anticancer Activity

The cytotoxicity profile of complexes **1** and **2** is compared to that of cisplatin (CDDP) and summarized in Table 1, listing the IC_50_ values from the MTT tests performed after 72 h of treatment. Among the tested 10 types of human cancer cell lines, 5 are suspension growing cells (the last five rows in Table 1), whereas all the others are the anchorage-dependent adherent type. We emphasized these cell characteristics, since a clear tendency could be seen in the obtained cytotoxicity of both studied complexes. The human embryonic kidney cell line (HEK-293) was used as a model for normal healthy cells in the estimation of cancer selectivity of the tested compounds. Moreover, two types of chemoresistant cell lines: HL-60/Dox and HL-60/CDDP were used as a measure for the potency of the studied complexes to circumvent the multidrug resistance of cancer cells. To present more clearly the superior toxicity of complex **1**, we also conveyed the calculated fold increase (FI) as a ratio of the IC_50_ values for cisplatin over that of complex **1** for each of the tested cell line. The resistance factor (RF) was calculated for each compound and was defined as the ratio of the IC_50_ values on the resistant derivative of the HL-60 line over that of the chemosensitive HL-60 cells.

The most obvious conclusion from the cytotoxicity data is that complex **1** exhibited nanomolar toxicity against most of the tested cell lines, which, in most cases, was two orders of magnitude higher than cisplatin (FI > 100). The exceptions were the REH and HT-29 cells, where the cytotoxicity of **1** was higher by only five and ten times, respectively. Most sensitive were the T-24, HL-60, HL-60/CDDP, and SKW-3 lines, with IC_50_ estimates < 100 nM after 72 h of exposure to complex **1**. Another general observation was that complex **2** showed lower or no detectable toxicity against the adherent cells, with the only exception being the T-24 and HT-29 cancer models. Notably, T-24 cells are of the adherently growing type that, in our screenings, showed a very high sensitivity to all the tested compounds, comparable, in some cases, to the sensitivity of the suspension cultures. Among all the tested cells, the colorectal carcinoma cell line (HT-29) was the least sensitive to complex **1**, with an estimated IC_50_ of only 3.5 µM, which, however, still surpassed cisplatin considerably. Apart from the mentioned exceptions, complex **2** was virtually non-toxic to the other adherent cancer cell lines of both malignant (CASKI, MDA-MB-23, and MCF-7 (IC_50_ > 200 µM)) and healthy (HEK-293) origins. 

Regarding the selectivity of cancer cells, a distinct behavior was seen for both studied complexes that depended on the highlighted differences in the cells’ growing characteristics. Namely, complex **1** showed no distinction between cancerous and healthy cells and was also equally toxic to chemosensitive and chemoresistant HL-60 cells. In contrast, complex **2** was virtually non-toxic to HEK-293 healthy cells, like the aforementioned adherent cancerous cells, whereas its toxicity to most of the suspension cells was comparable with cisplatin. Interestingly, among all the cells sensitive to complex **2**, the multidrug resistance derivatives of HL-60 (HL-60/Dox and HL-60/CDDP) were the most strongly affected. These general distinctions in the cytotoxicity profiles of complexes **1** and **2** might be explained by the previously discussed reactivity of the complexes against endogenous reducing agents.

### 3.3. Platinum Uptake and LDH Activity

**Figure 4:**
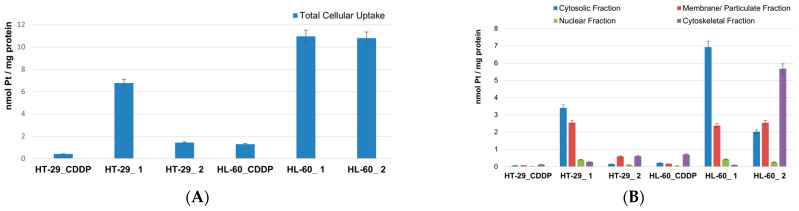
Pt uptake (in units nmol Pt/mg protein) by HL-60 and HT-29 cells after 4 h of incubation with 10 μM solutions of complexes **1**, **2**, and cisplatin (CDDP): (**A**) total platinum content in whole cells and (**B**) platinum content in cellular fractions: cytosolic, membrane, nuclear, and cytoskeletal.

In search of a plausible explanation for the observed trends in the estimated cytotoxicity of complexes **1** and **2**, we measured the platinum uptake by two types of cancer cells: HL-60 and HT-29 that are of suspension and adherent types, respectively. Commonly accepted units for presenting the results are the Pt content per 1 million cells or Pt content per µg of cellular protein. Since the tested cell lines significantly differed in their protein content and morphological properties, the data from the platinum uptake experiments are shown in units nmol Pt/mg protein to minimize the effects of the mentioned cellular characteristics. Numerical data from the performed ICP-MS measurements (in both unit types) and the measured protein contents can be found in the Supplementary Materials (Tables S1–S3). Except for the total platinum content in whole cells, the cellular fractions were also subjected to ICP-MS analyses. Figure 4A depicts the total platinum content in HL-60 and HT-29 cells after 4 h of incubation with 10 μM solutions of complexes **1**, **2**, and CDDP, and the platinum content in the different cellular fractions is shown in Figure 4B.

Among all the tested compounds, complex **1** showed the highest platinum uptake by HT-29 cells that was more than 10 times the uptake from the CDDP treatment and almost 5 times that of complex **2**. In the case of the HL-60 cells, the Pt uptake from the treatments with complexes **1** and **2** were virtually the same and were almost 10 times that of the CDDP treatment. In all cases, the suspension growing HL-60 cells was more prone to platinum uptake. This is in line with the observed higher sensitivity of HL-60 to the cytotoxic effect of the tested compounds, but the total platinum content alone could not fully convey the general conclusions from the cytotoxicity analyses. The data obtained on the preferential platinum accumulation in different cell fractions bring additional insight into the cellular distribution and compartmentalization of the substances upon their internalization in the cells. Our findings clearly show that, irrespective of the cell type, complex **1** accumulates predominantly in the cytosolic fraction, whereas complex **2** remains trapped in the cytoskeletal fraction. The second-most abundant in platinum was the membrane/particulate fraction for both complexes and in both cell types. The deepest and hardest to reach cellular compartment represented by the nuclear fraction was reasonably least platinated after the treatment period with the tested compounds, given the short 4 h exposure time.

Along with the uptake measurements, we checked the supernatants of the treated cells for enzymatic activity, specifically lactate dehydrogenase (LDH), as an indirect measure for preservation of the cellular membrane during the incubation. The results showed that the LDH activity in the supernatants of all the treated cells remained virtually the same as the control samples (Figure S27 in the Supplementary Materials). This should indicate that cell membrane destruction was not the cause for the observed antitumor activity of the studied compounds.

### 3.4. Proteome Analyses

**Figure 5:**
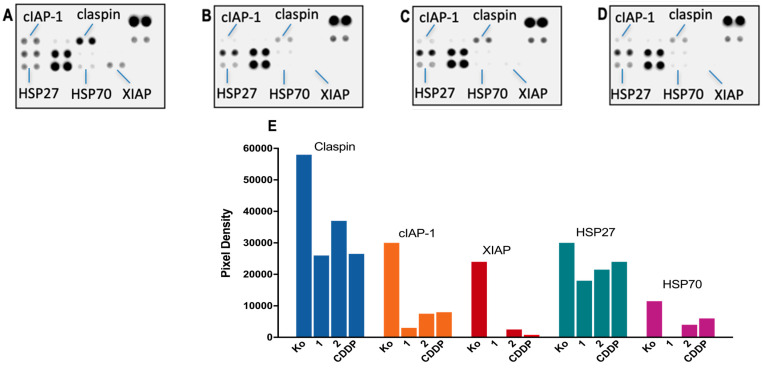
Changes in expression levels of apoptosis-related proteins in HL-60 cells following treatment with complex **1** (**B**); complex **2** (**C**); CDDP (**D**) as compared to the untreated control—Ko (**A**). Cells were exposed to equi-effective concentrations (IC_50_) of the metal complexes for 24 h, following which, a human proteome profiler assay was performed. A further densitometric analysis of the array spots was conducted using ImageJ 1.8.0 software, and the most prominent changes in the proteome were expressed graphically (**E**).

The proapoptotic activity of the studied complexes was assessed in a proteome analysis of HL-60 cells using cisplatin (CDDP) as a reference drug. Thereby, cellular transcriptomic changes in key apoptosis-related proteins were tracked following 24 h exposure to the metal complexes (Figure 5). 

None of the treated samples showed cleavage and activation of the effector pro-caspase 3; however, significant alterations were found in the expression levels of several regulatory factors, including claspin, cIAP-1, XIAP, and the chaperones HSP27 and HSP70. Furthermore, a consistent trend was observed in the degree of proteomic changes induced by the metal complexes, where the strongest modulation exhibited complex **1** (with complete disappearance of the spot signals for XIAP and HSP70), followed by **2** and CDDP. 

A nearly two-fold reduction in the claspin levels, a key checkpoint regulator in the ATR–claspin–Chk1 replication pathway, was observed in response to the treatment with **1**. There was also a substantial weakening of the spot signals for the survival factors cIAP-1 and XIAP that function as caspase inhibitors and preclude apoptotic cell death at an earlier stage. Modulation in some components of the chaperone network was also established, where all metal complexes produced a moderate decline in HSP27 and HSP70 heat shock proteins that promote cell survival in response to cytotoxic stimuli. 

## 4. Discussion

To the best of our knowledge, this is the first report on the anticancer activity of a platinum(IV) complex bearing a polycyclic aromatic side arm. Among most appreciated drug design guidelines, the axial ligand of choice should be biologically active by itself, either as a drug or a modulator of certain cellular biochemical networks related to cell survival and proliferation. Although pyrene belongs to the class of polyaromatic hydrocarbons (PAH) that are notorious for their toxicity [41], some structural similarities between the pyrenebutyrate fragment used by us and approved drugs that have been utilized as axial ligands for Pt(IV) prodrugs (e.g., phenylbutyrate, naproxen, and carprofen) can be speculated. Moreover, pyrene is a very good intercalator and has been used in numerous biological studies, either in the design of potent DNA intercalators [42] or as a guest molecule for probing host–guest interactions with other aromatic systems [34,43,44,45]. Our recent cytotoxicity data on pyrene itself demonstrated that it has no detectable toxicity at concentrations up to 100 µM [34]. Similar conclusions have been reported on some pyrenebutyrate derivatives as well [44,45]. In earlier studies, pyrenebutyrate was employed as a hydrophobic counter ion to mediate the cytosolic delivery of large therapeutic peptides through membrane translocation or through the cell binding of large quantum dots [46,47]. From our studies on modulating the cytotoxicity of highly selective metallosupramolecular capsules by the encapsulation of aromatic guest molecules, pyrene proved its ability to turn the capsules non-toxic and thereby make them suitable host–guest delivery systems selective toward cancer cells [34,35]. Herein, we attached the intercalating pyrenebutyrate as a pendant arm of the Pt(IV) complexes that can be readily liberated upon reduction of the complex and eventually release the cytotoxic Pt(II) species inside the cancerous cell. The pyrenebutyrate Pt(IV) complexes were isolated in good yields and purity by employing two different synthetic strategies: coupling agent TBTU was used for the disubstituted complex **2**, and the NHS-activated ester of 1-pyrenebutyric acid was used to obtain complex **1**. The structures of the complexes were confirmed by multinuclear, 1D, and 2D NMR spectra and IR spectroscopy (all shown in the Supplementary Materials). In line with the purpose of our design, the first point of this investigation was the reactivity of complexes **1** and **2** with bioavailable reductants. Among the tested reducing agents, only ascorbic acid could completely reduce complex **1** for less than two days, whereas complex **2** was only partially reduced for three days (Figure 2). This is not surprising, due to the presence of a relatively more activated OH ligand in complex **1** as compared to complex **2**. The better reactivity of the monosubstituted Pt(IV) complex agrees with observations from other studies [9,48], although the roles of the equatorial ligands should not be underestimated [7,8]. We could identify by ^195^Pt NMR the new Pt(II) species that were formed from the reduction of complex **1** as DMSO complexes with characteristic signals at ca. −3030 and −3050 ppm (Figure 3B). The two signals most probably correspond to different coordination modes of the DMSO molecules in the formed complexes, which is an ambidentate ligand that can coordinate either with the S or the O atom. A detailed analysis on the possible Pt(II) complexes with DMSO and their NMR shielding of the Pt nuclei was provided earlier by other authors [40]. Unlike ascorbic acid, the 1 e^−^-reductant glutathione could not cause a reduction of the complexes, even after more than 10 days in our experimental conditions. Such observations have been reported for other similar complexes of Pt(IV) [28,48]. The acidity of the medium might be a crucial factor in the expected two-electron reduction of Pt(IV). Hamilton et al. demonstrated the role of acidity on the reduction and the resulting cytotoxicity of oxoplatin and its derivatives by comparing the in vitro antiproliferative activity of the compounds if pretreated or not with 0.1 M HCl [49]. The results showed that, while cisplatin’s cytotoxicity was not affected by the presence of HCl, that of oxoplatin and its derivatives was strongly increased when they were pretreated with 0.1 M HCl. Such an effect was only slightly detected for the bis-acetato derivative of oxoplatin they studied. These data support the activation-by-reduction hypothesis on the anticancer activity of Pt(IV) prodrugs and are in line with our reactivity and cytotoxicity data on complexes **1** and **2**. 

The observed differences in the reactivity of complexes **1** and **2** with ascorbic acid correlate very well with our cytotoxicity data (Table 1). They indicate the very high cytotoxicity of complex **1**, whereas **2** is virtually non-toxic to most of the adherent type of cancerous cells, as well as to the healthy cells, HEK-293. Indeed, complex **2** shows a high toxicity (in the low µM range) only to the multidrug-resistant cells HL-60/CDDP and HL-60/Dox. The expected increased levels of glutathione efflux proteins in the resistant cells might facilitate alternative reduction mechanisms or catalyze the reduction caused by ascorbic acid. The high propensity for the reduction of complex **1** reflects its cytotoxicity that, for several types of cancer cells, is in the nM range; specifically, the IC_50_ was estimated at 50 to 70 nM against most of leukemic cells and 60 nM against the T-24 urinary bladder carcinoma cell line. These toxicity levels are more than two orders of magnitude higher than cisplatin, in some cases. Specifically, a very high toxicity was observed against the multidrug-resistant derivatives of the HL-60 parental line. These data suggest a notable difference in the antiproliferative mechanism of action of the studied complexes **1** and **2** as compared to cisplatin. Our previous data on the toxicity level of pyrene against four types of cancer cells [34] are also included in Table 1, whereas the cytotoxicity of oxoplatin was extensively studied in a panel of 38 human cancer cells by Hamilton et al. [49]. A notable drawback of complex **1** is its complete lack of selectivity to cancer cells, since the survival of healthy cell model line HEK-293 is equally well inhibited. This observation is in contrast with some of our earlier studies on the ligand substitution kinetics of the Pt(II) and Pd(II) supramolecular systems [33] or water-soluble Pt(IV) complexes [50], which suggested a higher selectivity for the more labile complexes. One should note, however, that, in the mentioned ligand substitution reactions, the complexes also changed their charge that might affect the fate of the formed species inside the cell, which was not the case for the reduction of the studied Pt(IV) complexes. 

Apart from the reactivity of the complexes, their ability to penetrate the cell membrane and successfully internalize is a key step for the discussed biological activity. Our platinum content measurements revealed a 10-fold higher uptake for both complexes in leukemic cells HL-60 as compared to cisplatin (Figure 4A and Table S1 in the Supplementary Materials). The observed much higher platinum uptake by HL-60 cells as compared to HT-29 cells is in line with the known larger accessibility of the suspension cells’ membranes for xenobiotics than those of the adherent cells growing on monolayers. Interestingly though, complex **1** penetrates both types of cells almost equally well, whereas complex **2** shows a distinct inability to accumulate into adherent HT-29 cells in contrast to its high uptake by HL-60 cells. An even more enhanced uptake of platinum is achieved from treating the adherent HT-29 cells with complex **1** as compared to cisplatin. The increased uptake of slightly less lipophilic monosubstituted complexes is usually ascribed to active transport mechanisms [23]. As several authors have reported, however, an apparent correlation between lipophilicity, cellular accumulation, and cytotoxicity could not be established [10,23,25,39]. In our case, the significant degree of internalization of complex **1**, both in the suspension and adherent cells, clearly correlated with its cytotoxicity. For complex **2**, however we observed a deviation from this simple trend; the equally enhanced total uptake in HL-60 cells did not result in an increased cytotoxicity as compared to cisplatin, which was in sharp contrast to the trend seen for complex **1**. Similarly, the higher total platinum uptake in HT-29 cells treated with complex **2** did not reflect on its cytotoxicity as compared to cisplatin. A close look at the platinum accumulation in the cellular fractions pointed to very different fates of the internalized complexes **1** and **2**. Complex **2**, like cisplatin, accumulated in the cytoskeletal fraction, whereas complex **1** was predominantly found in the cytosolic fraction. Although these results could not allow for drawing specific conclusion related to the mechanism of action, it was clearly seen that complex **1** exhibited distinct accumulation and a cytotoxicity profile with very high cytotoxicity to all type of cells. Destruction of the cellular membrane as a stray and nonspecific cause for cell death was excluded as a possible mechanism based on our LDH activity measurements in the supernatants of the treated cells, which showed virtually the same results as the control cells (Figure S27 in the Supplementary Materials). To gain insight into the eventual effects of the tested compounds on the protein expression, the most chemosensitive cells, HL-60, were subjected to a proteome analysis. This involved a series of immunoassay experiments to follow the changes in 24 different apoptotic and survival signaling proteins. Although none of the complexes indicated alterations in the procaspase 3 levels, three regulatory factors: claspin, cIAP-1, and XIAP were significantly inhibited by complex **1**. Claspin is a known regulatory factor involved in the cellular response to DNA damage and replication stress and is needed to ensure the faithful replication and repair of damaged DNA [51]. On the other hand, cIAP-1 and XIAP belong to the group of apoptosis protein inhibitors that play a crucial role in the regulation of apoptosis. cIAP-1 suppresses the activity of caspases, which are proteases responsible for executing the cell death process; similarly, XIAP is the X-linked inhibitor of apoptosis protein that is potent for inhibiting caspase-3, -7, and -9, all critical mediators of apoptosis. The estimated significant decrease in the expression of these two proteins, caused by all the tested compounds but more profoundly by complex **1**, is the most probable reason for the caspases to become activated and initiate the apoptotic cascade [52]. Another pair of proteins with decreased expression levels are the chaperones HSP27 and HSP70 that are heat shock proteins, which assist in protein folding, prevent protein aggregation, and aid in the refolding of denatured proteins under stress conditions. Often, they work in cooperation, and their chaperone activities are essential for cell survival under adverse conditions [53]. Our data showed that, similar to XIAP, the HSP70 protein was completely knocked down by the treatment with complex **1**. These two groups of proteins that regulate the complex mechanisms of cell apoptosis and survival suggest pathways of cellular intervention of the tested complexes **1** and **2** that could be related to the observed cytotoxicity. 

## 5. Conclusions

Unlike the well-established design of Pt(IV) prodrugs, we utilized 1-pyrenebutyric acid as an axial ligand in continuation of our works with pyrene-encapsulating metallosupramolecular hosts and in search of cytotoxic pyrene-bearing guest molecules. The studied mono- and disubstituted Pt(IV) analogs of cisplatin: complexes **1** and **2**, showed rather distinct cytotoxicity profiles on the tested 10 types of cancerous cell lines. While complex **1** was highly cytotoxic, with IC_50_ values in the nanomolar range to most cell lines, complex **2** exhibited a high toxicity only to the multidrug-resistant derivatives of the HL-60 cells (HL-60/CDDP and HL-60/Dox). Notably, complex **2** showed limited toxicity to the tested carcinoma cell lines and was practically non-toxic to the healthy cells, HEK-293. In contrast, complex **1** was devoid of selectivity to cancerous cells. These differences could be related to the distinct kinetics towards reduction with ascorbic acid that was evidenced by the NMR measurements; the reduction of complex **1** was completed in two days, whereas complex **2** was only partly reduced in three days. Apparently, the supposed activation-by-reduction mechanism was operative for the cytotoxicity of the studied Pt(IV) complexes, and its kinetics was at the basis of their selectivity. Both studied complexes showed a significant enhancement in platinum uptake as compared to that of cisplatin, though their fates after cellular internalization were rather distinct; complex **1** accumulated in the cytosol, whereas **2** was trapped in the cytoskeletal fraction. A proteome analysis on the most sensitive HL-60 cells identified two groups of regulatory proteins significantly inhibited by the complexes, especially by complex **1**. These are the proteins from the family of apoptosis protein inhibitors (API) and heat shock proteins responsible for regulating cell survival. Given all these data, one could expect that the ongoing chemical transformations of the complexes in the cytosol are the strongest factor that correlates with the observed cytotoxicity of the compounds. We can suppose that the reactivity of the complexes and their ability to accumulate inside the cells are the two characteristics that can be chemically fine-tuned to better adjust the selectivity of these complexes. Moreover, pyrene’s high propensity for intercalation and other supramolecular interactions gives room to modulate the selectivity to cancer cells of the more reactive complex **1** through its encapsulation and targeted delivery by suitable nanomaterials. The biological activity of bis-pyrenebutyrate complex **2** also deserves more attention for elucidating the specific protein interactions that could provide a highly efficient and selective way to overcome multidrug resistance of cancer cells.

## Figures and Tables

**Table 1:** Cytotoxicity data for complexes **1** and **2** obtained from the MTT test after 72 h of treatment and presented as IC_50_ values (in µM). Cisplatin (CDDP) is used as a reference cytostatic compound. The resistance factor (RF), defined as the ratio of the IC_50_ values on the resistant derivative of the HL-60 line over that of the chemosensitive HL-60 cells, is shown in parentheses. The fold increase (FI) in the cytotoxicity is calculated for **1** as the ratio of IC_50_ values obtained for CDDP over that of complex **1**.

Cell Line	CDDP	Complex 1	Fold-Increase (FI)	Complex 2	Pyrene
HEK-293 ^a^	13.8 ± 3.3	0.2 ± 0.1	69	n.d. (>200)	-
CASKI ^b^	16.2 ± 1.8	0.15 ± 0.06	108	n.d. (>200)	-
MDA-MB-231 ^c^	48.3 ± 3.1	0.7 ± 0.2	69	n.d. (>200)	-
MCF-7 ^d^	55.5 ± 4.3	0.3 ± 0.15	185	n.d. (>200)	-
HT-29 ^e^	36.6 ± 1.5	3.4 ± 0.7	10.8	90.0 ± 10.2	>100 *
T-24 ^f^	10.4 ± 0.8	0.06 ± 0.01	173	4.9 ± 1.1	>100 *
HL-60 ^g^	9.8 ± 1.1	0.07 ± 0.01	140	18.1 ± 3.9	>100 *
HL-60/Dox ^h^	32.9 ± 1.2	0.1 ± 0.01	329	2.2 ± 0.5	>100 *
**RF**	**3.4**	**1.4**		**0.12**	
HL-60/CDDP ^i^	135.2 ± 2.9	0.05 ± 0.01	2704	1.9 ± 0.8	-
**RF**	**13.8**	**0.7**		**0.06**	
REH ^j^	1.1 ± 0.8	0.2 ± 0.03	5.5	3.1 ± 0.9	-
SKW-3 ^k^	8.3 ± 0.9	0.06 ± 0.01	138	9.8 ± 2.0	-

^a^ Human embryonic kidney cells; ^b^ cervical cancer cell line; ^c^ triple-negative breast cancer cell line; ^d^ hormone-dependent breast cancer cell line; ^e^ colorectal carcinoma cell line; ^f^ urinary bladder carcinoma cell line; ^g^ promyelocytic leukemia cell line; ^h^ multi-resistant promyelocytic leukemia cell line; ^i^ CDDP-resistant promyelocytic leukemia cell line; ^j^ precursor B-lymphoblastic leukemia cell line; ^k^ acute T-lymphoblastic leukemia cell line; n.d.—not detectable; *—data from [34].

## Data Availability

The data presented in this study are available upon request from the corresponding author.

## Supplementary Materials

The following supporting information can be downloaded at https://www.mdpi.com/article/10.3390/pharmaceutics15092310/s1: Figures S1–S26. Spectral studies on the structures and reactivities of the complexes; Tables S1–S3. Numerical data from the Pt-uptake studies and total protein contents in the treated cancer cells; Figure S27. the LDH activity data in the supernatants of the HL-60 and HT-29 cells after 4 h of incubation with 10 μM solutions of complexes **1**, **2**, and cisplatin (CDDP).
