# Supplementary material for: Pyrenebutyrate Pt(IV) Complexes with Nanomolar Anticancer Activity
Source: Pharmaceutics. 2023 Sep 13;15(9):2310. doi: 10.3390/pharmaceutics15092310 (PMC10537052; doi:10.3390/pharmaceutics15092310)
Supplement: Supplementary file 1 [file pharmaceutics-15-02310-s001.zip › pharmaceutics-2597900-supplementary.pdf]

# Supplementary Materials to

## Pyrenebutyrate Pt(IV) Complexes with Nanomolar Anticancer Activity

**Anife Ahmedova [1,*], Rositsa Mihaylova [2], Silviya Stoykova [1], Veronika Mihaylova [1], Nikola Burdzhiev [1], Viktoria Elincheva [2], Georgi Momekov [2] and Denitsa Momekova [2]**

[1] Faculty of Chemistry and Pharmacy, Sofia University, 1, J. Bourchier Blvd., 1164 Sofia, Bulgaria; sstoykova@chem.uni-sofia.bg (S.S.); ahvm@chem.uni-sofia.bg (V.M.); ohnb@chem.uni-sofia.bg (N.B.)
[2] Faculty of Pharmacy, Medical University-Sofia, 2 Dunav Street, 1000 Sofia, Bulgaria; rmihaylova@pharmfac.mu-sofia.bg (R.M.); gmomekov@pharmfac.mu-sofia.bg (G.M.); dmomekova@pharmfac.mu-sofia.bg (D.M.)
* Correspondence: ahmedova@chem.uni-sofia.bg; Tel.: +359-2-8161-247

## Content

IR Spectra of all synthesized compounds ............................... 2

NMR Spectra of all synthesized compounds .......................... 4

Reactivity of the studied complexes with biological reductants followed by NMR ............................................................ 11

Platinum uptake data and LDH activity ............................... 15

# IR Spectra of all synthesized compounds

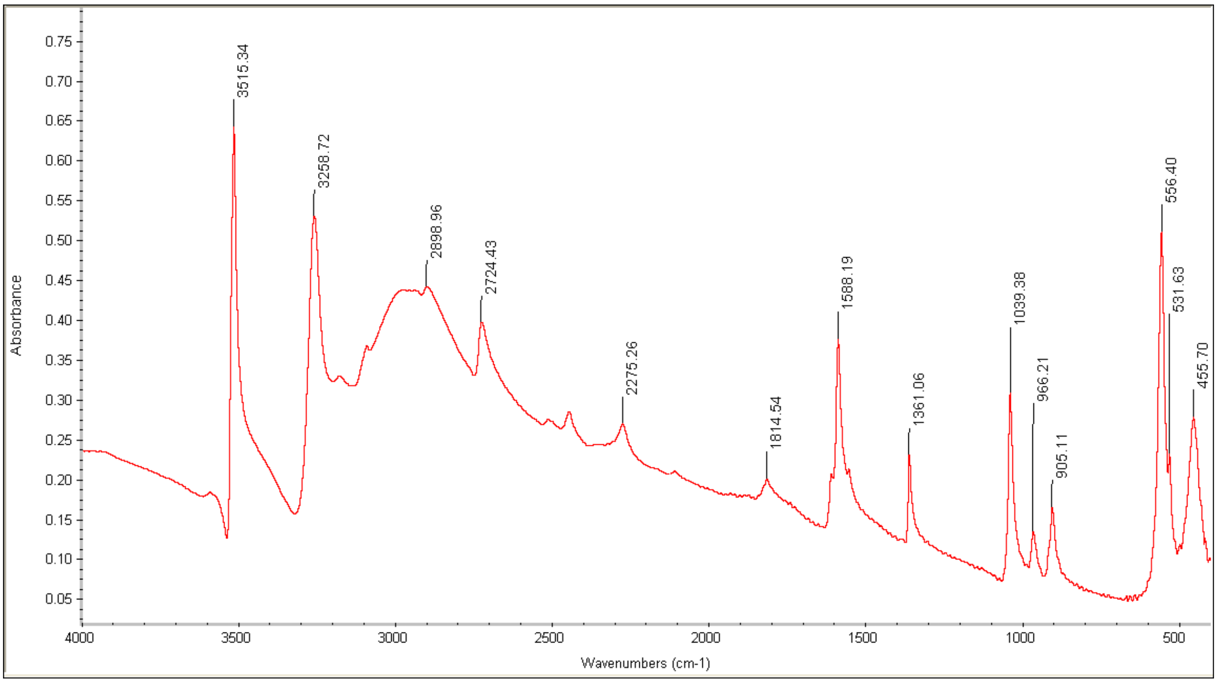


**Figure S1.** IR spectrum of oxoplatin, *c,c,t*-[Pt($NH_3$)$_2$$Cl_2$(OH)$_2$]

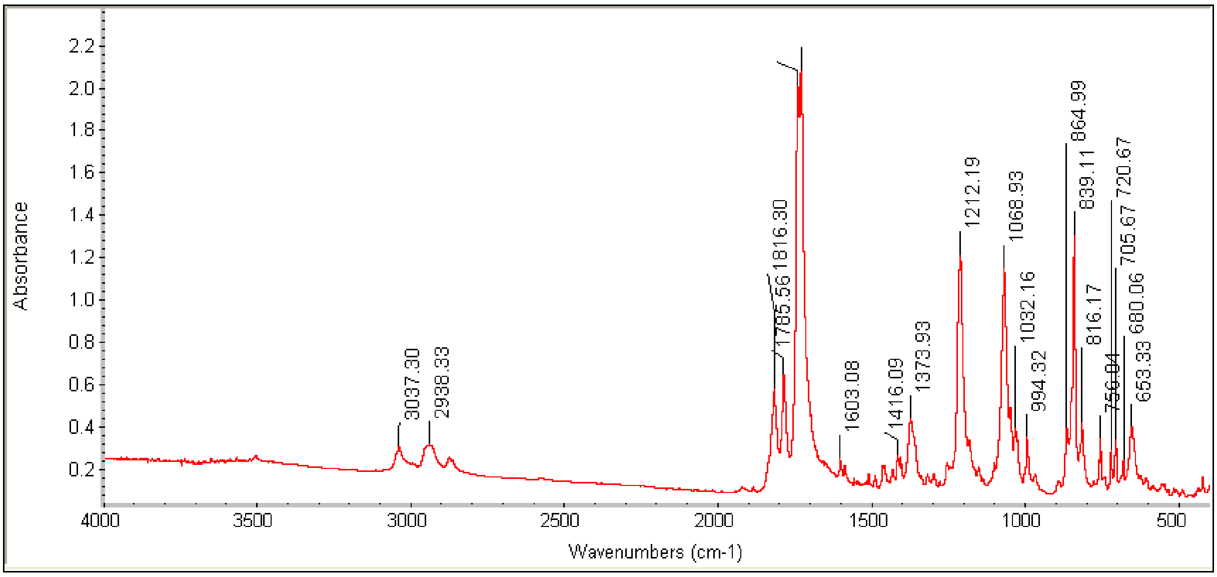


**Figure S2.** IR spectrum of activated NHS-ester of 1-pyrenebutyric acid.

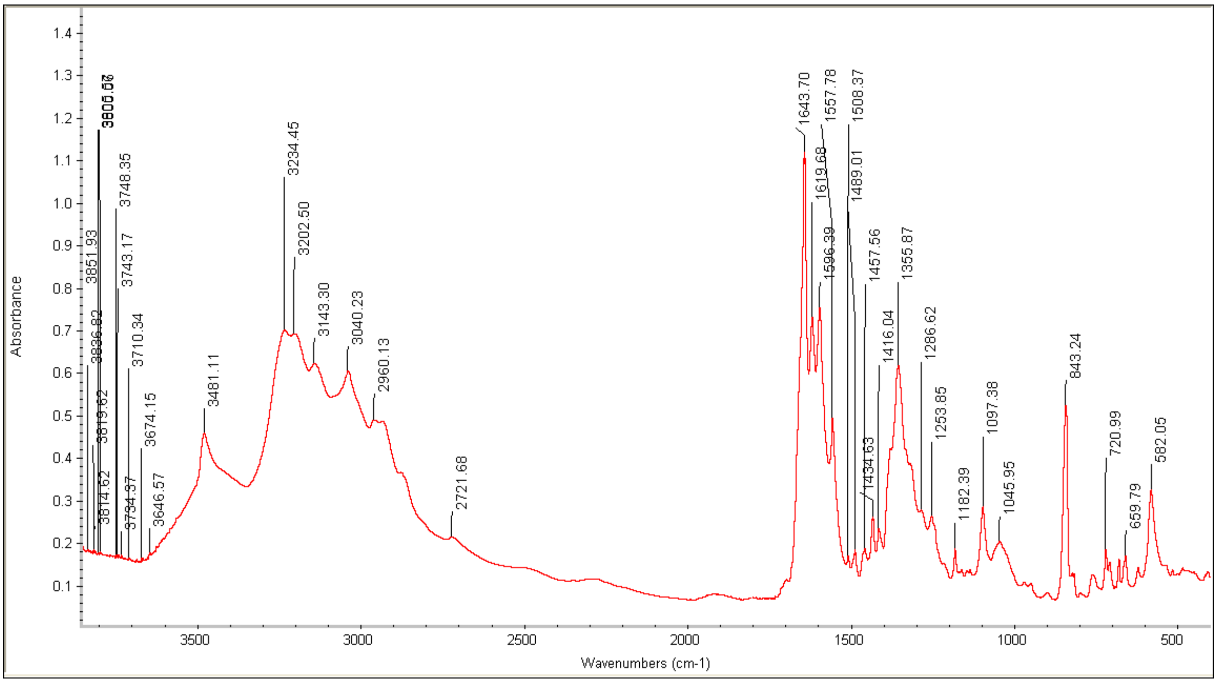

**Figure S3.** IR spectrum of complex **1**.

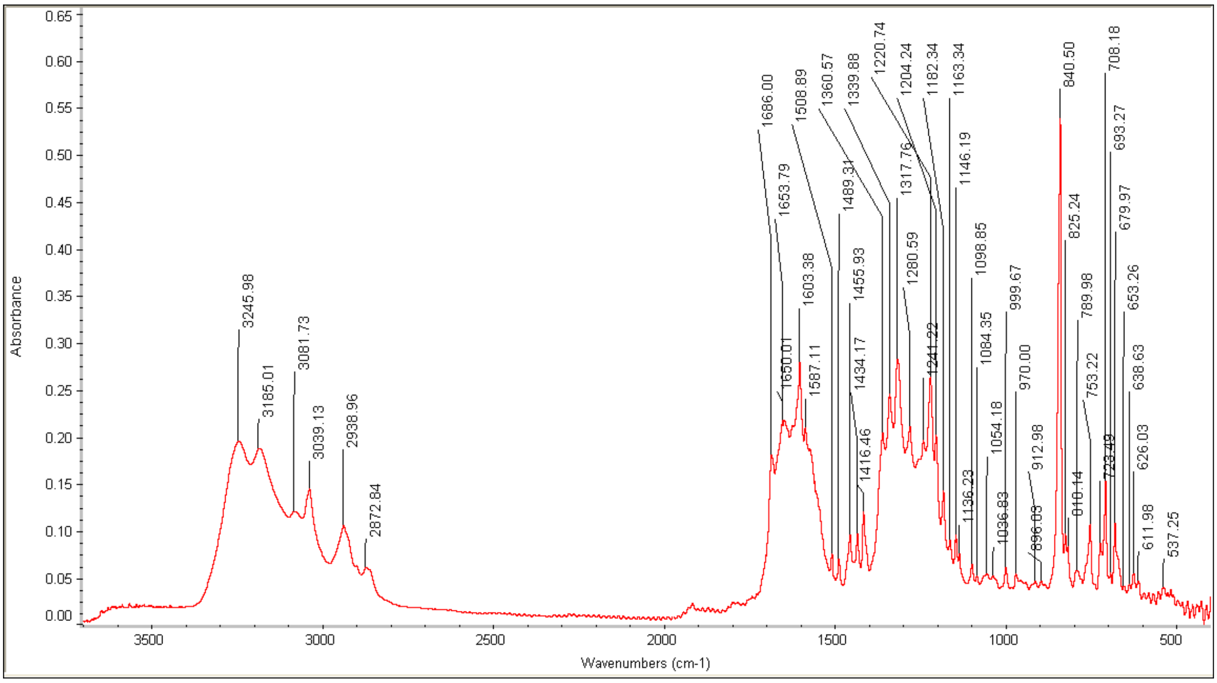

**Figure S4.** IR spectrum of complex **2**.

# NMR Spectra of all synthesized compounds

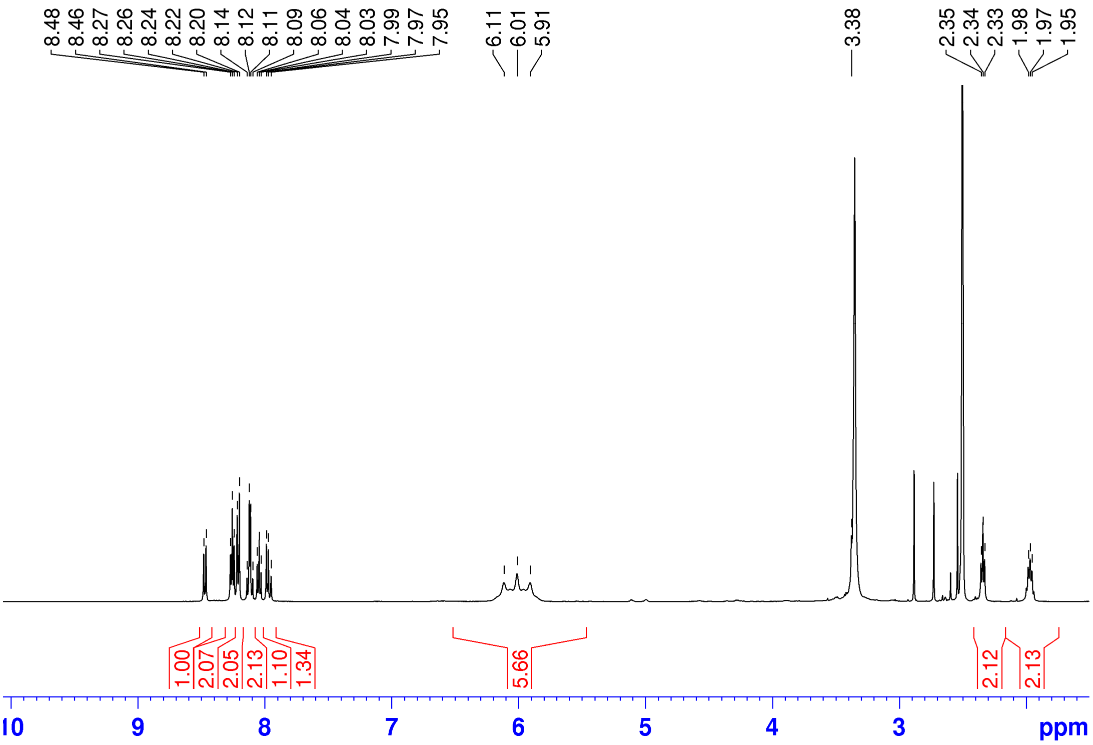


**Figure S5.** $^{1}H$ NMR spectrum of complex **1**.

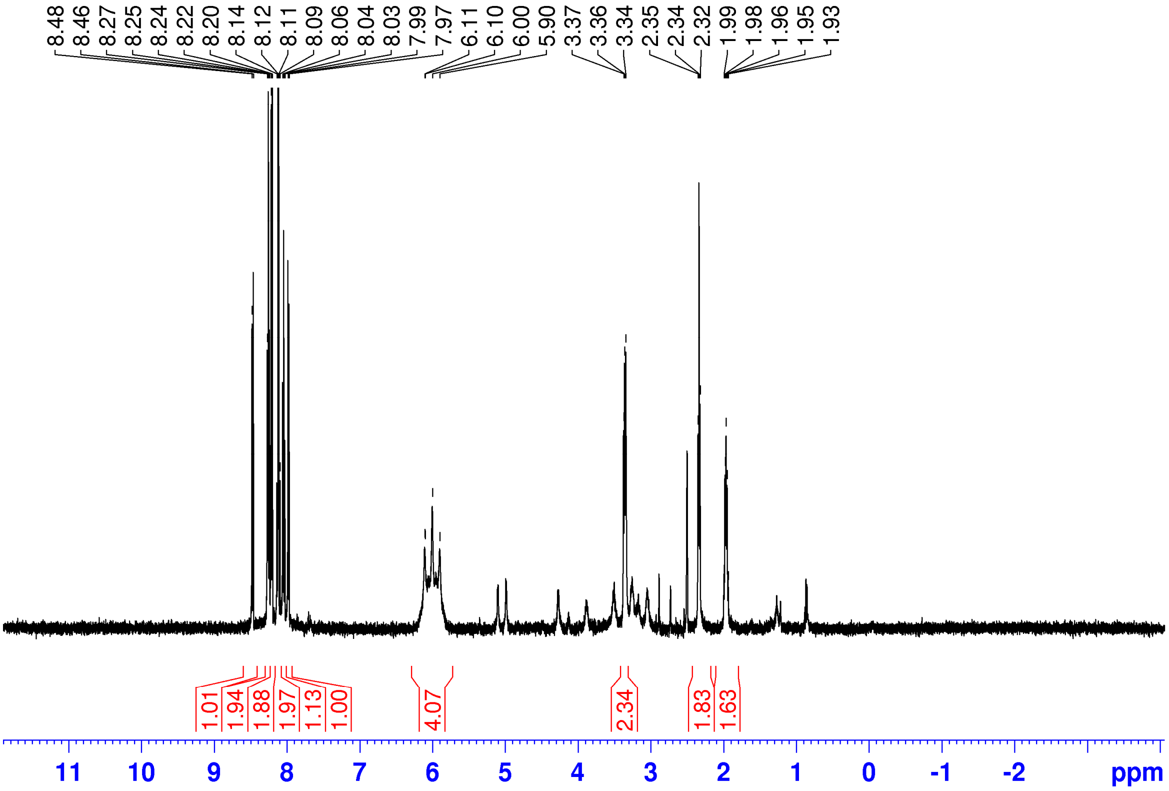


**Figure S6.** 1D DOSY NMR spectrum of complex **1**.

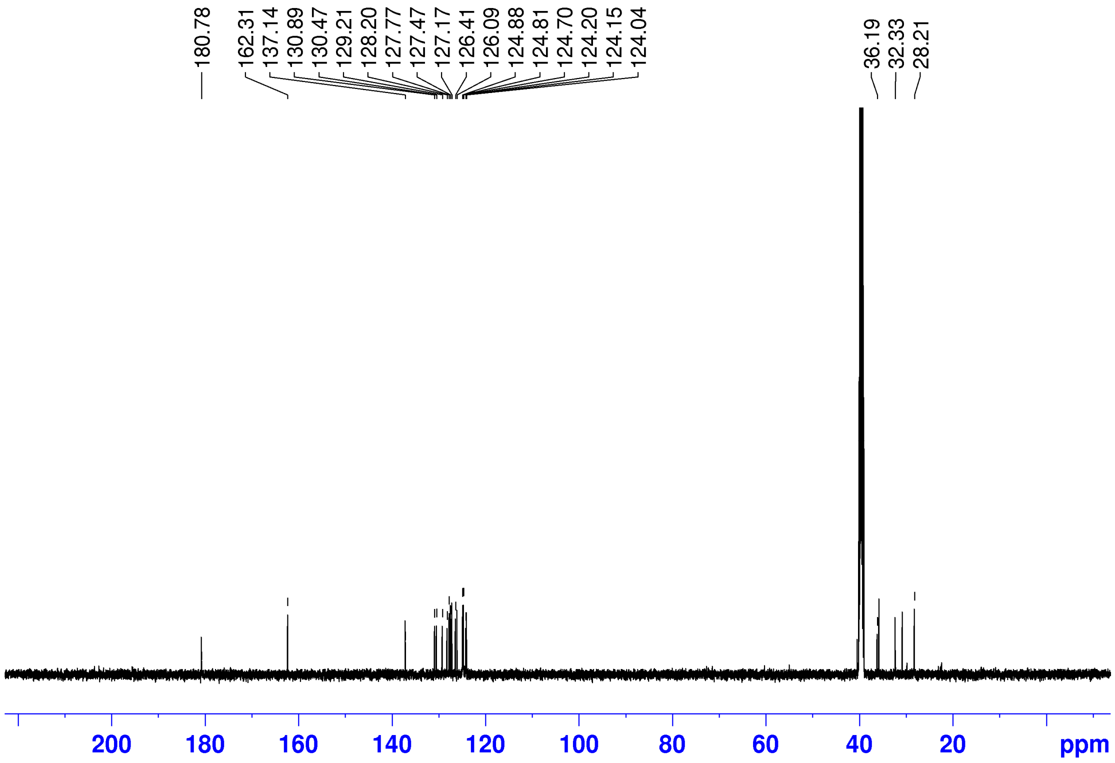


**Figure S7.** $^{13}C$ NMR spectrum of complex **1**.

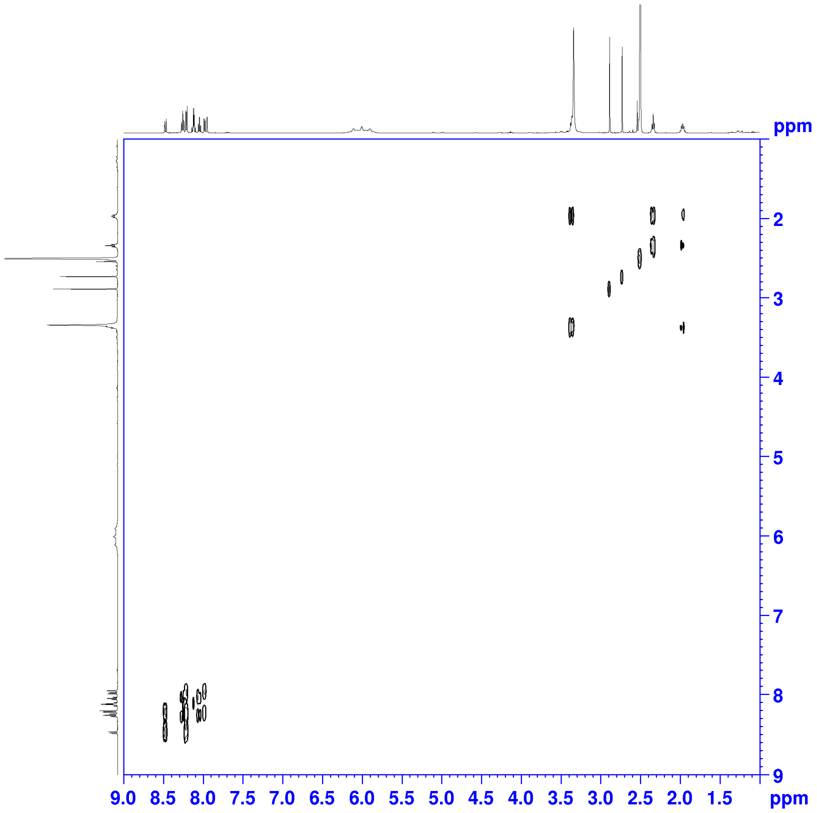


**Figure S8.** COSY NMR spectrum of complex **1**.

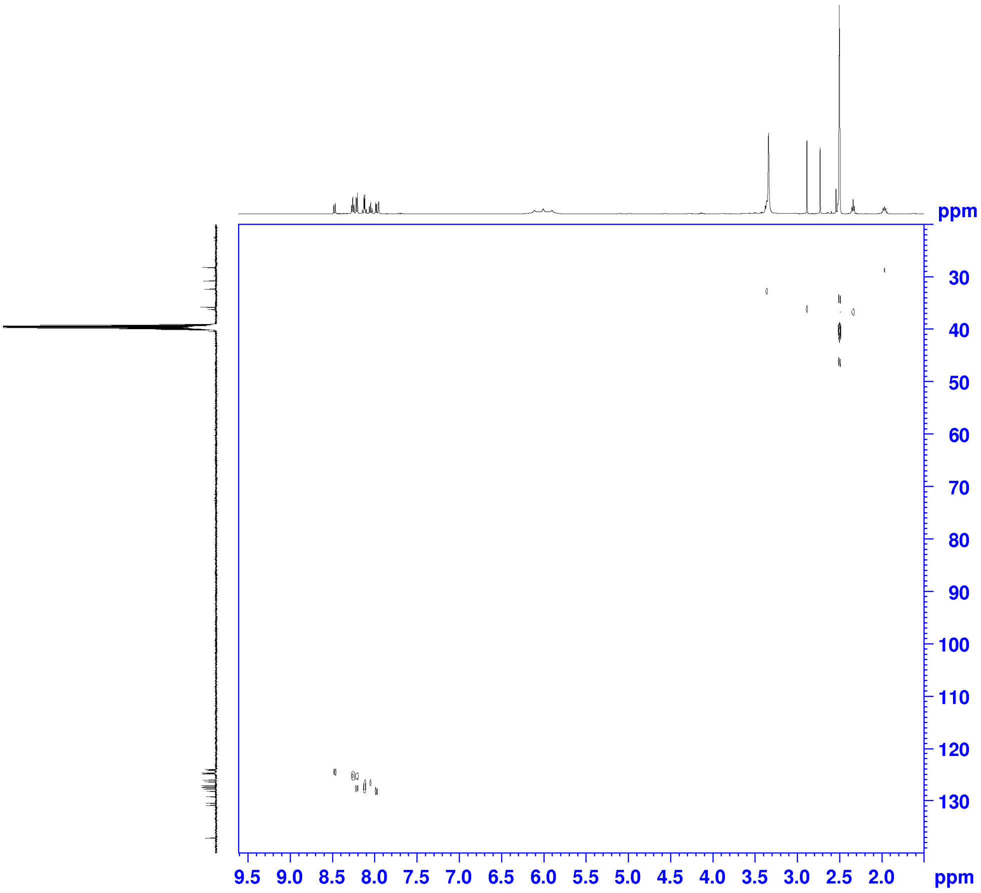


**Figure S9.** HSQC NMR spectrum of complex **1**.

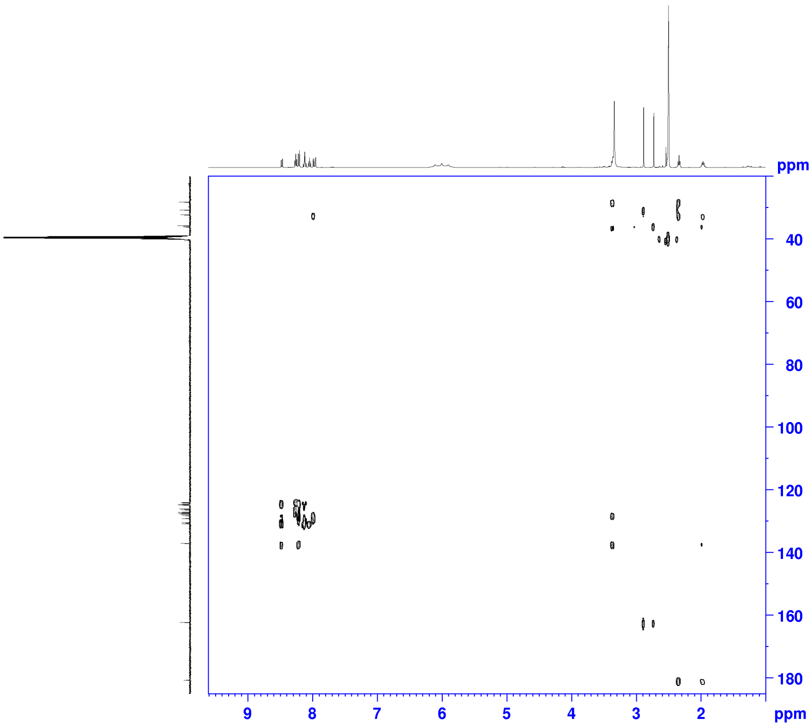


**Figure S10.** HMBC NMR spectrum of complex **1**.

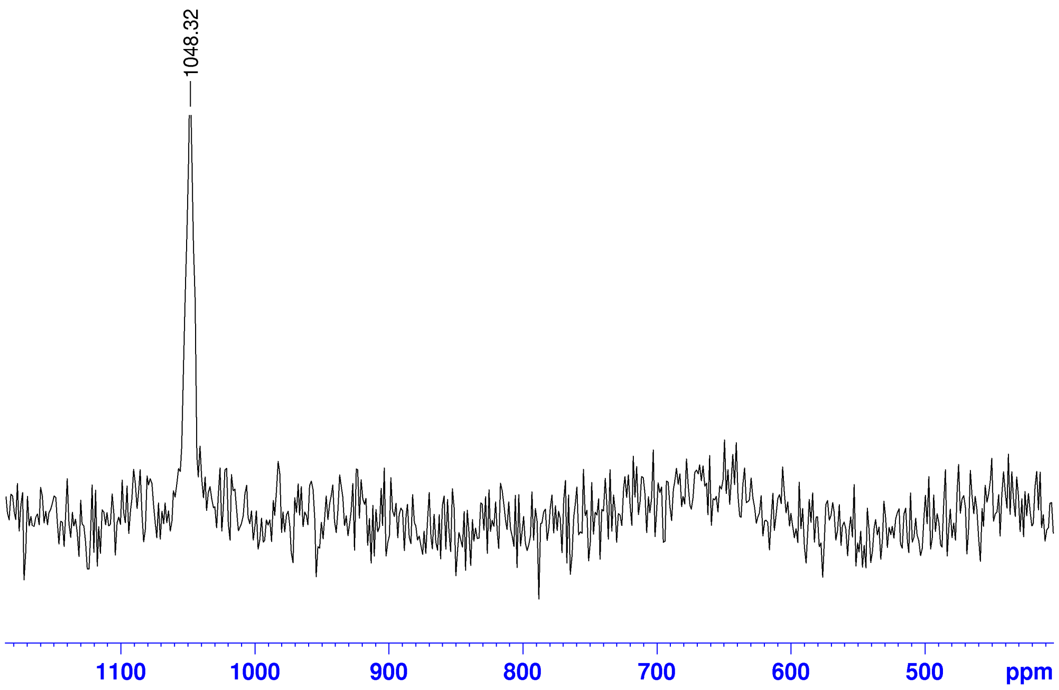


**Figure S11.** $^{195}$Pt NMR spectrum of complex **1**.

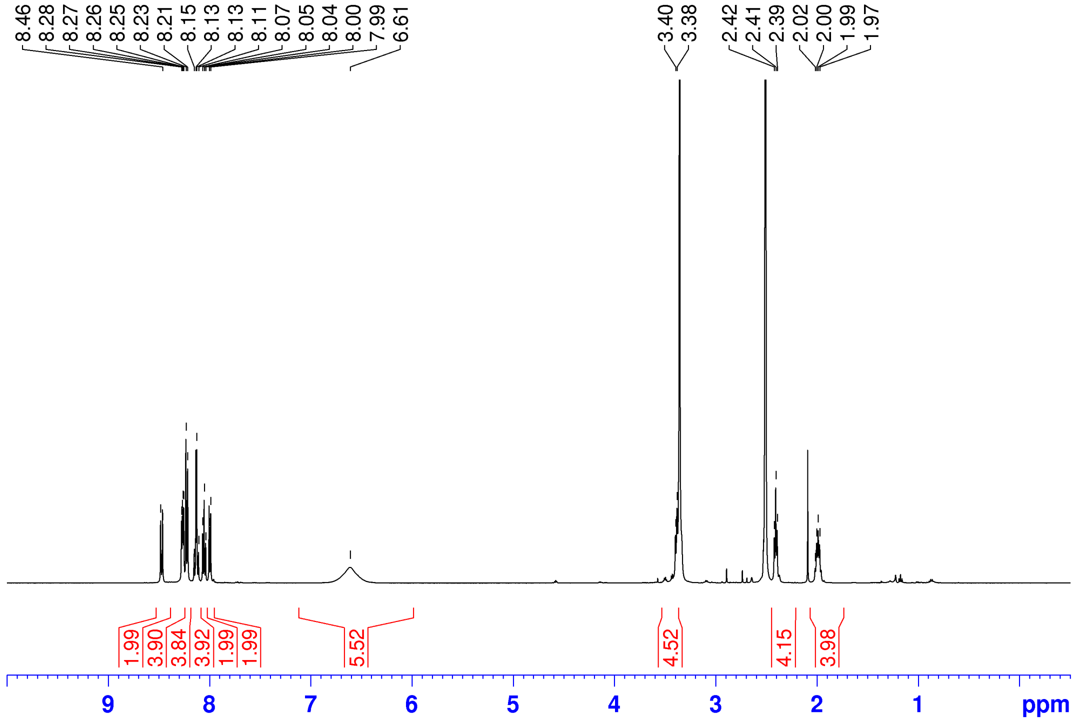


**Figure S12.** $^{1}$H NMR of complex **2**.

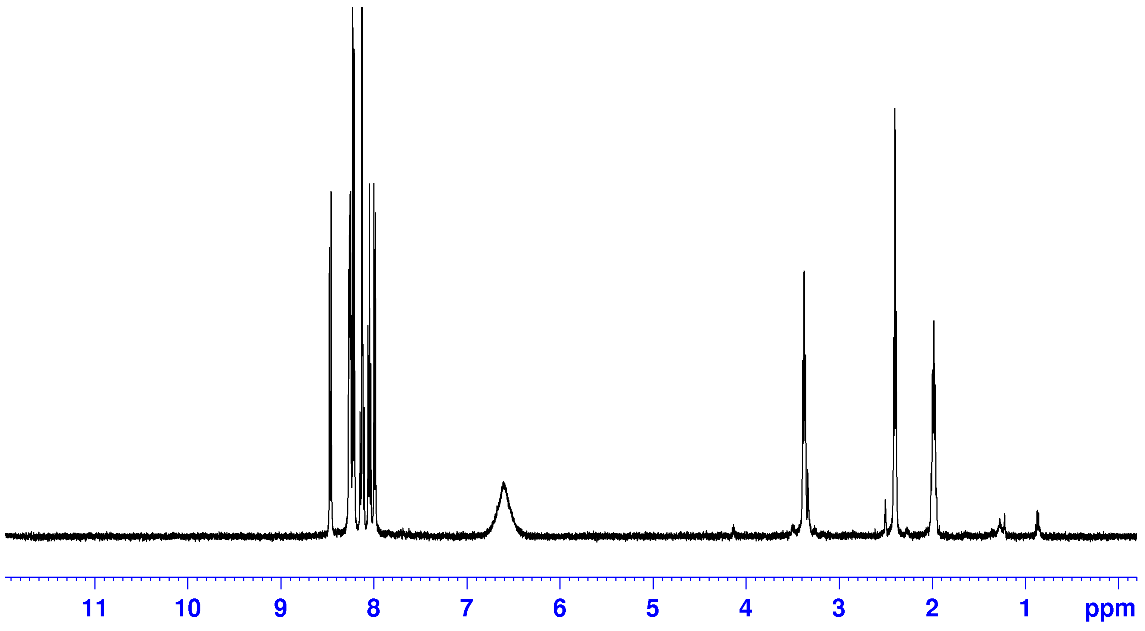


**Figure S13.** 1D DOSY NMR of complex **2**.

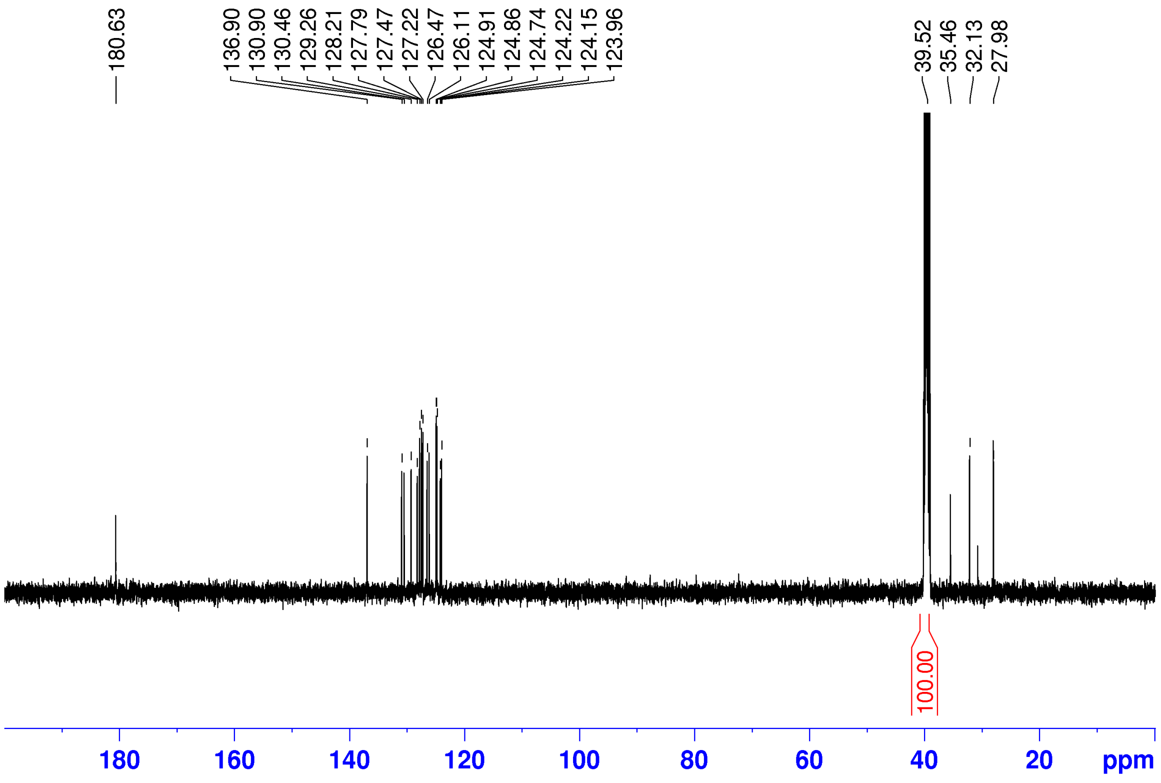


**Figure S14.** $^{13}C$ NMR of complex **2**.

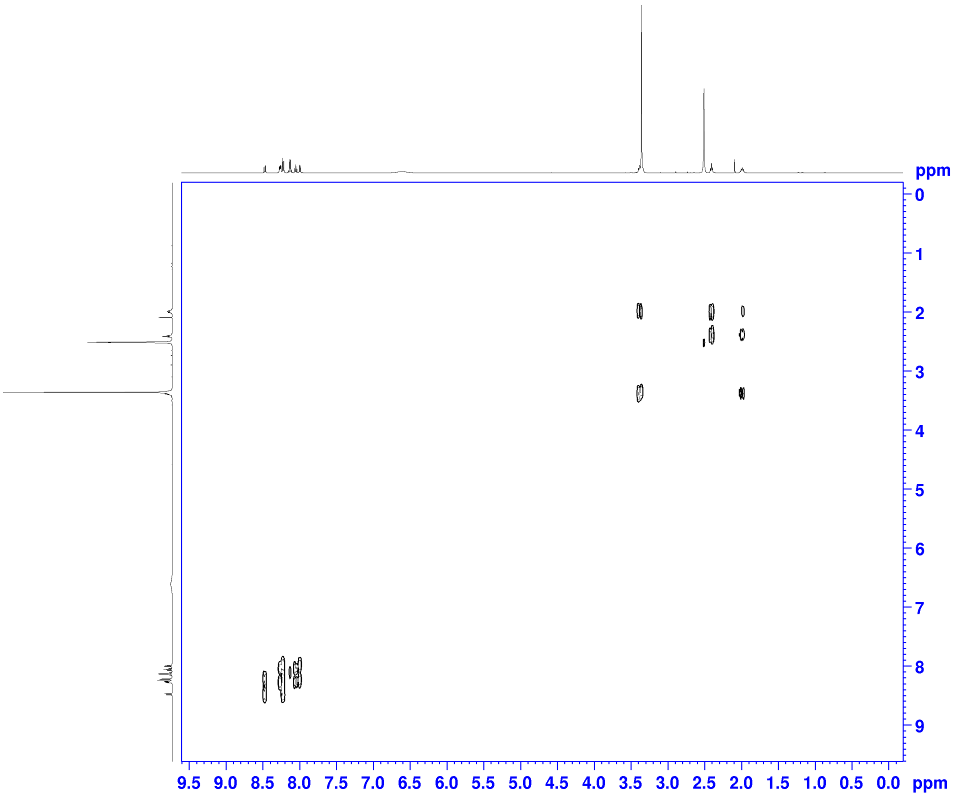


**Figure S15.** COSY NMR spectrum of complex **2**.

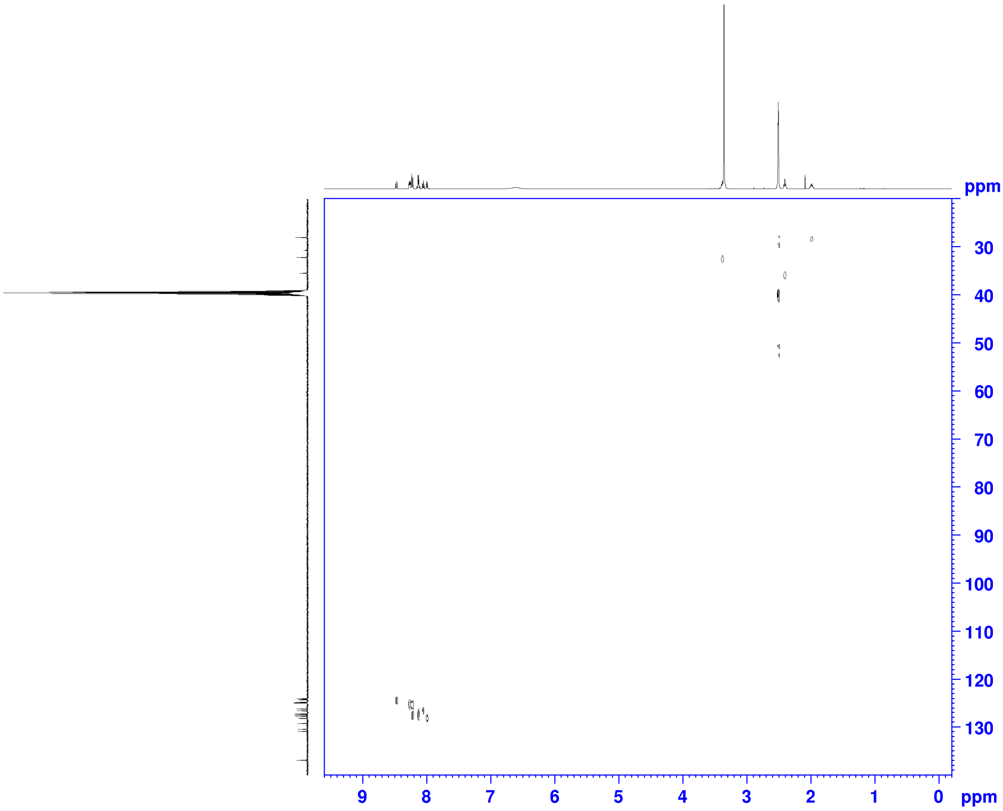


**Figure S16.** HSQC NMR spectrum of complex **2**.

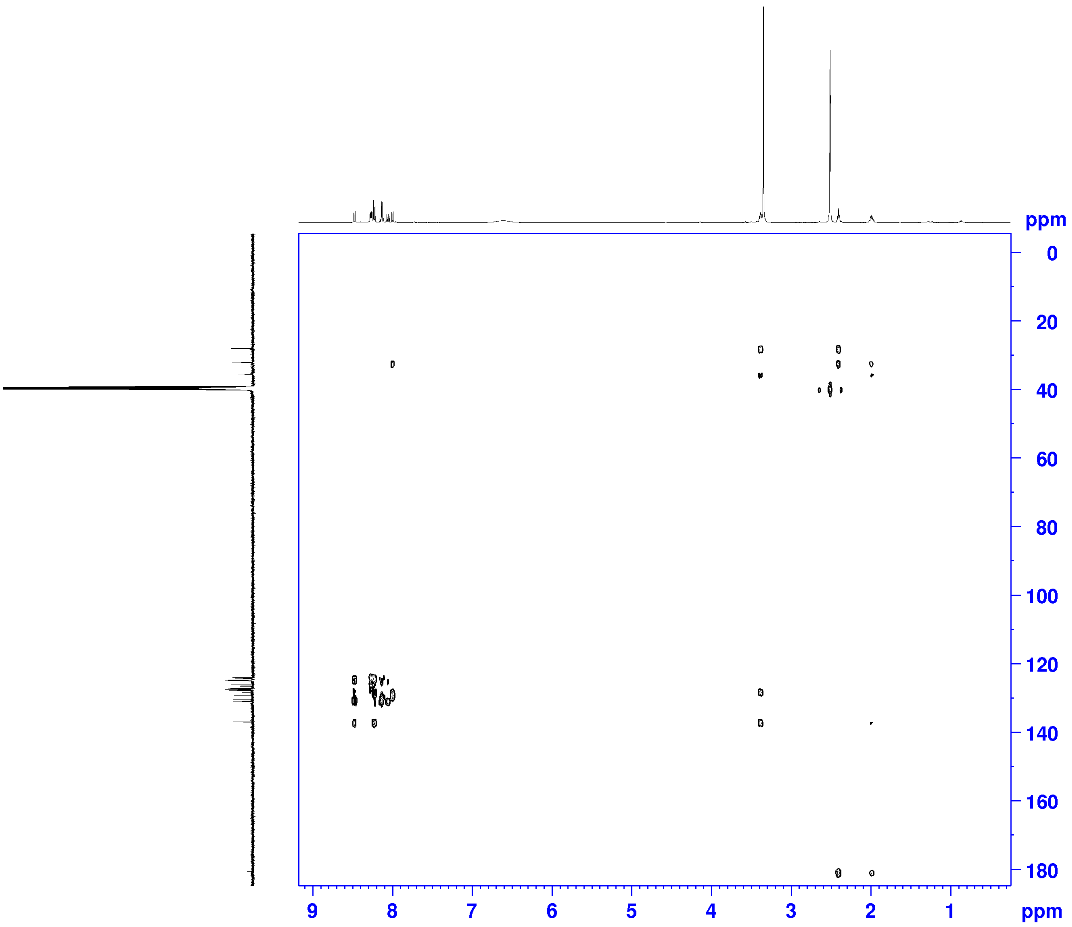


**Figure S17.** HMBC NMR spectrum of complex **2**.

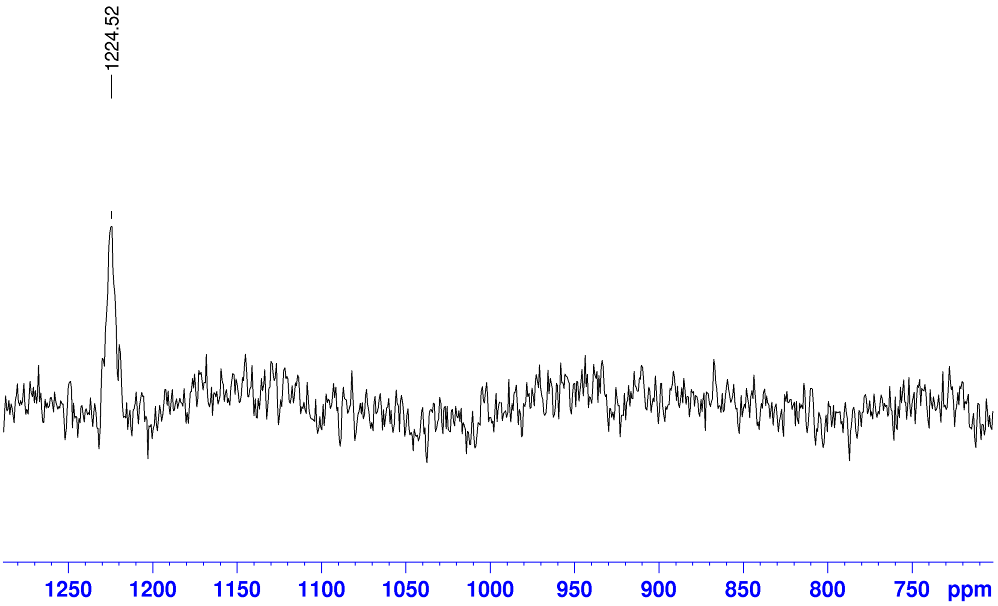


m

**Figure S18.** $^{195}$Pt NMR of complex **2**.

## Reactivity of the studied complexes with biological reductants followed by NMR

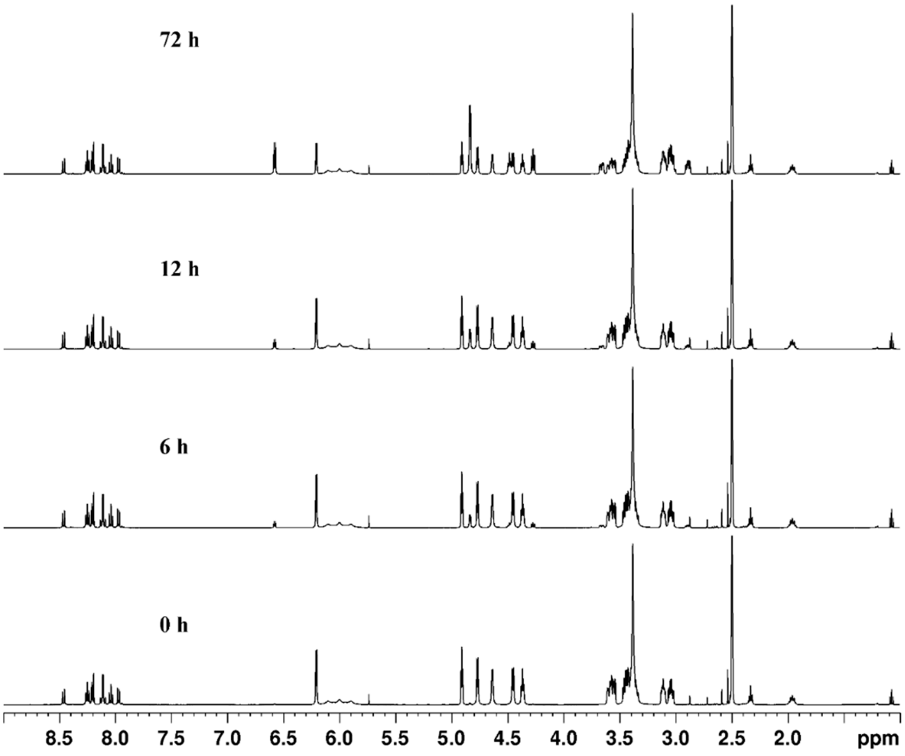


**Figure S19.** $^{1}H$ NMR spectra of complex **1** upon addition of glucose.

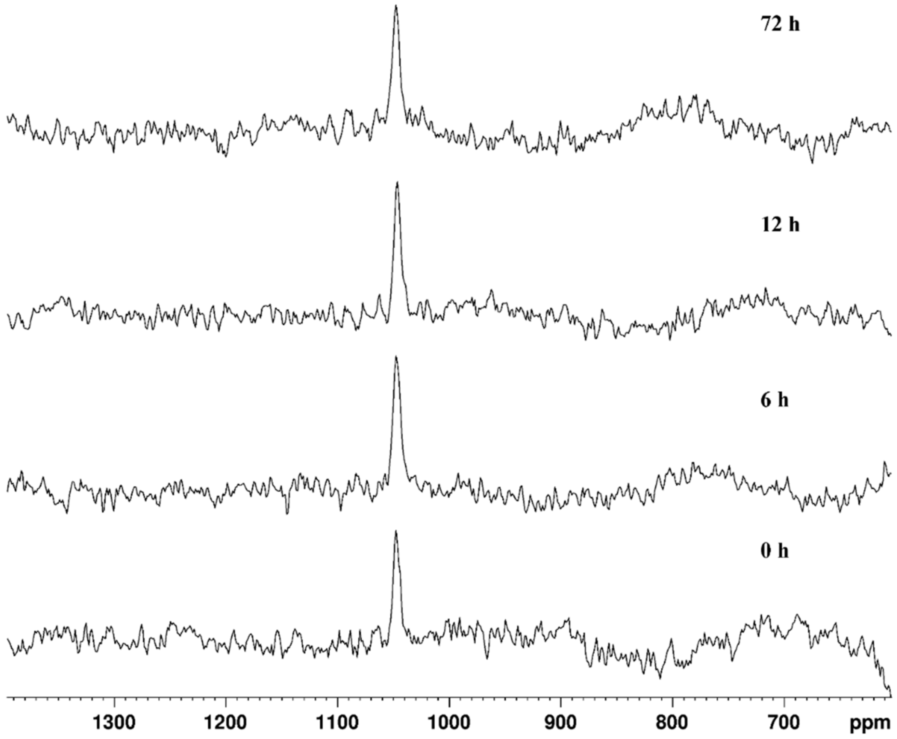


**Figure S20.** $^{195}Pt$ NMR spectra of complex **1** upon addition of glucose.

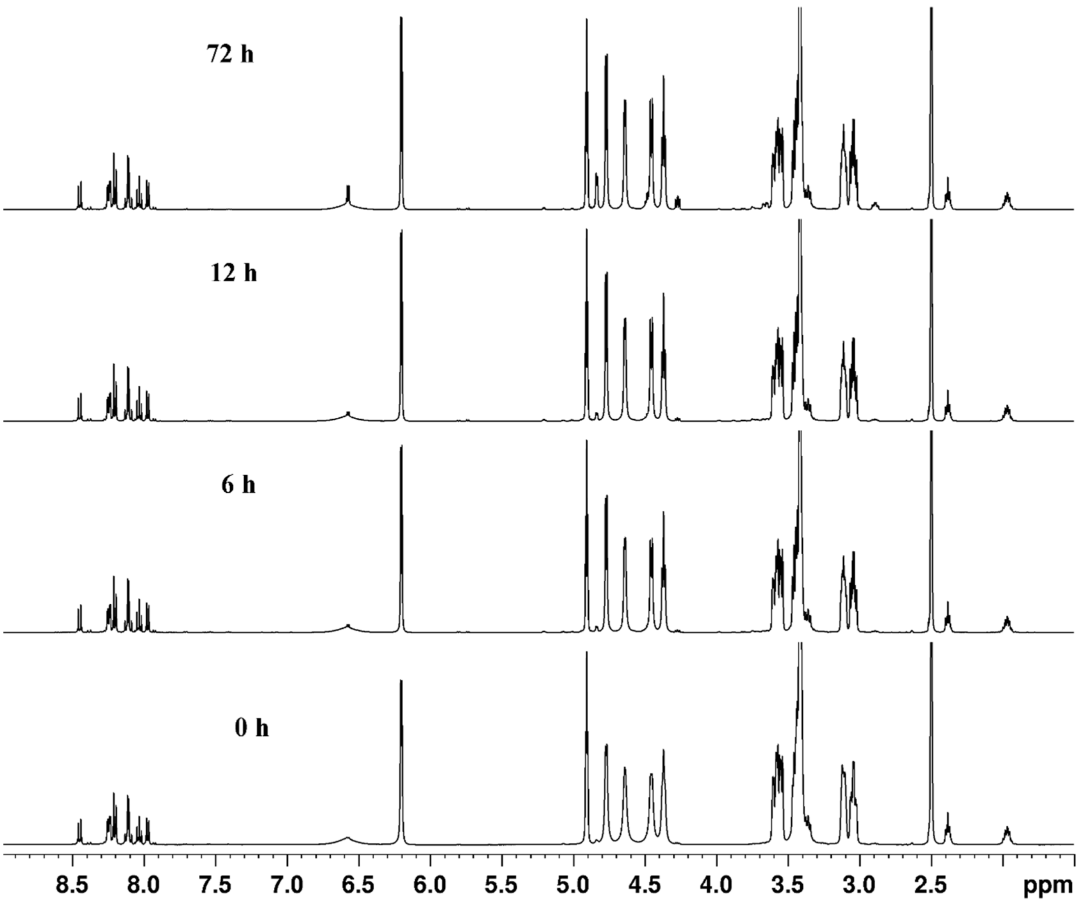


**Figure S21.** $^{1}H$ NMR spectra of complex **2** upon addition of glucose.

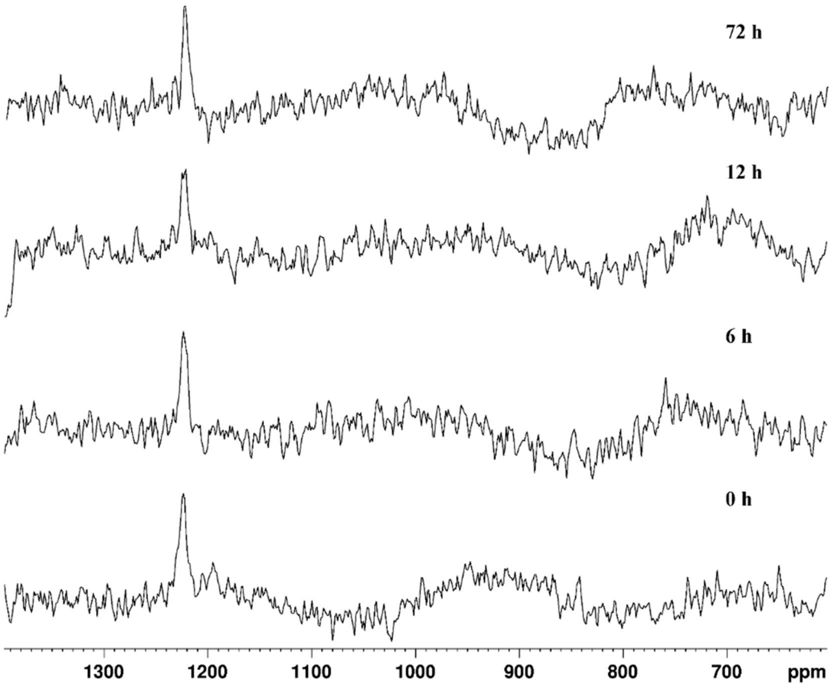


**Figure S22.** $^{195}Pt$ NMR spectra of complex **2** upon addition of glucose.

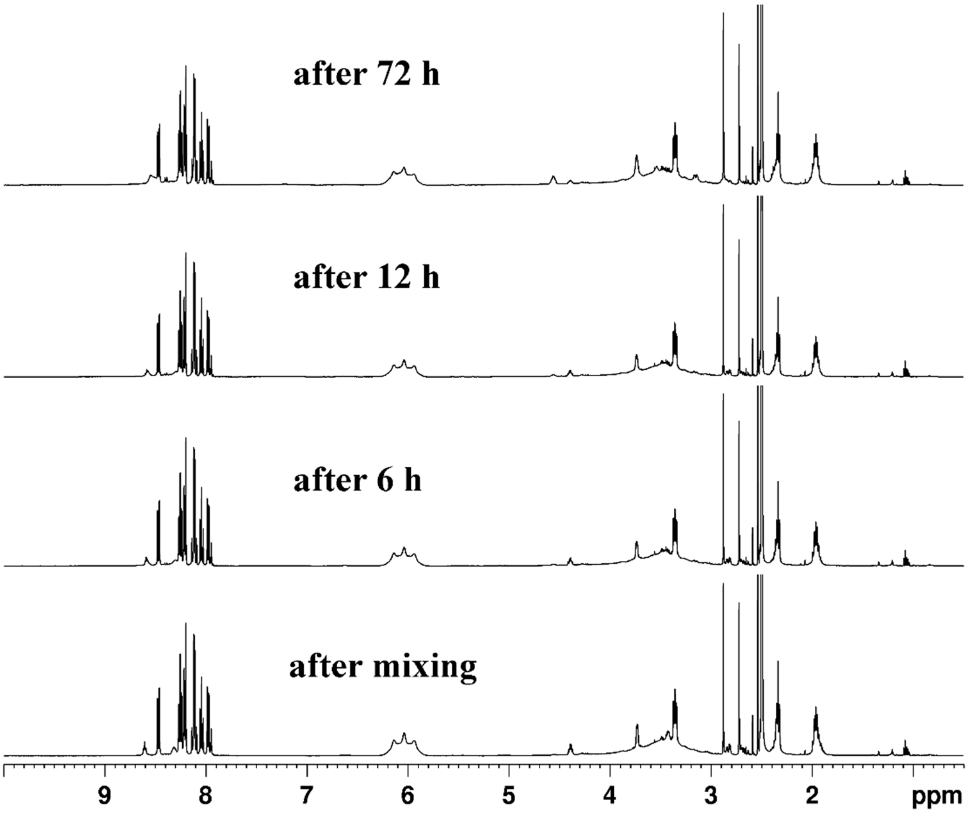


**Figure S23.** $^{1}H$ NMR spectra of complex **1** upon addition of glutathione .

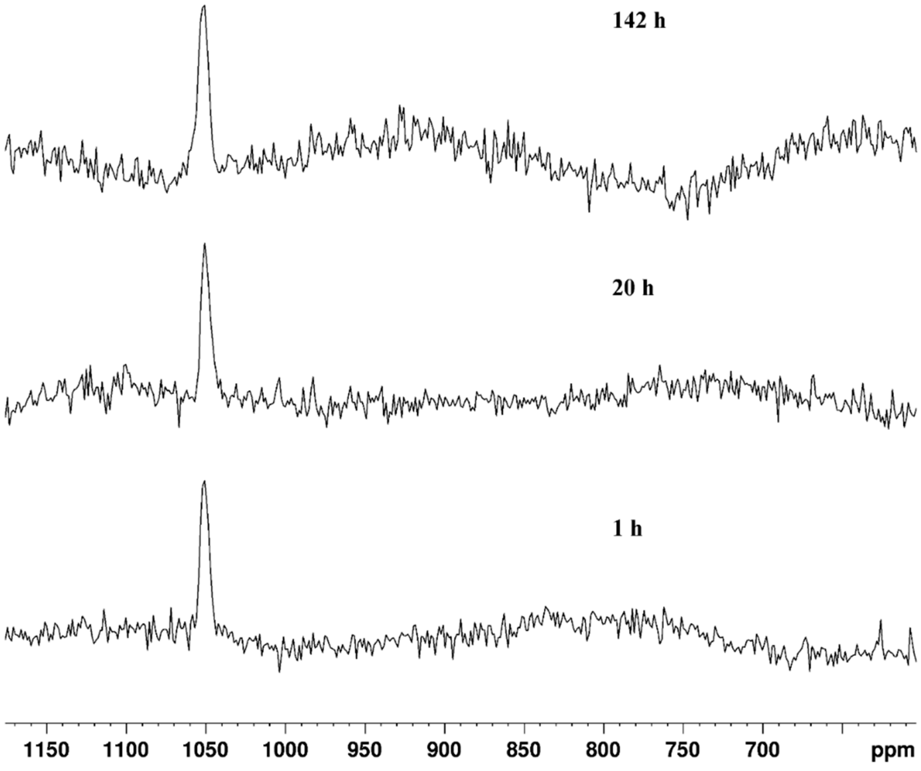


**Figure S24.** $^{195}Pt$ NMR spectra of complex **1** upon addition of glutathione.

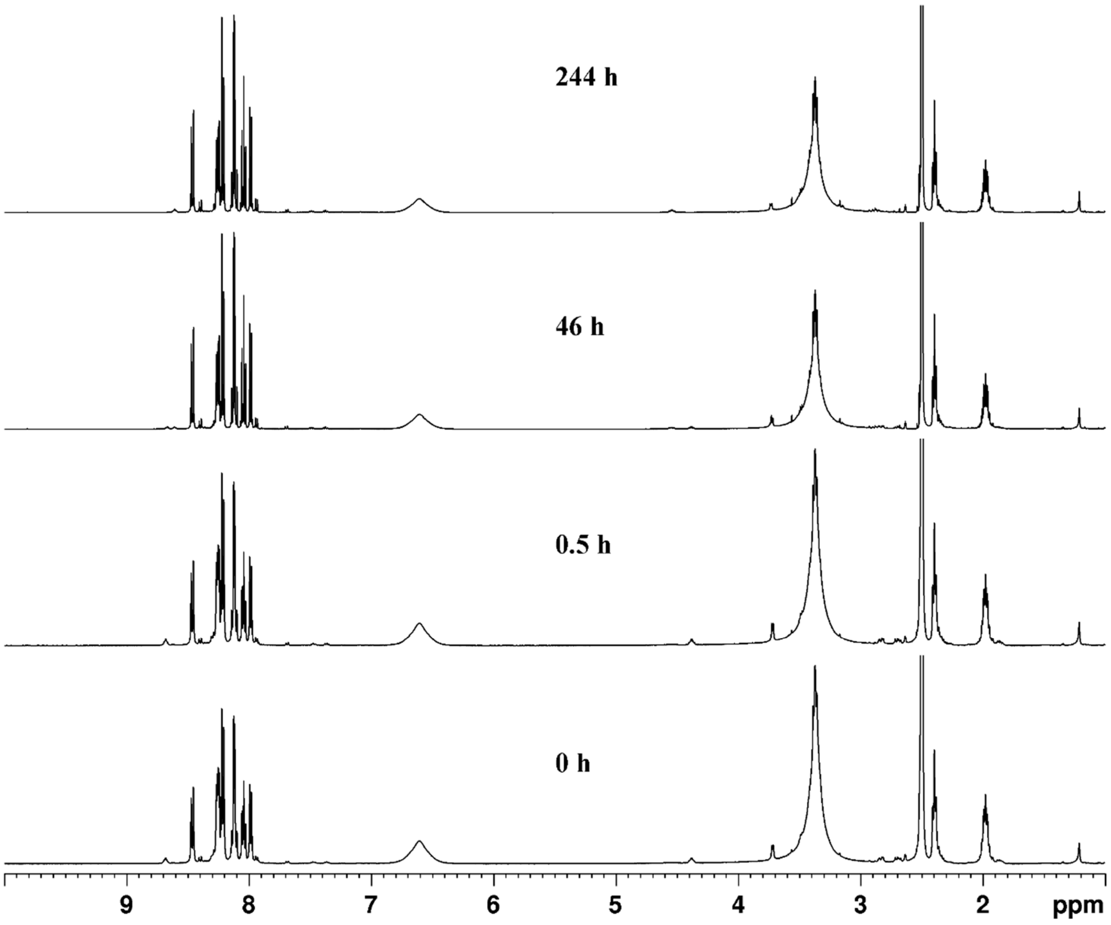


**Figure S25.** $^{1}H$ NMR spectra of complex **2** upon addition of glutathione.

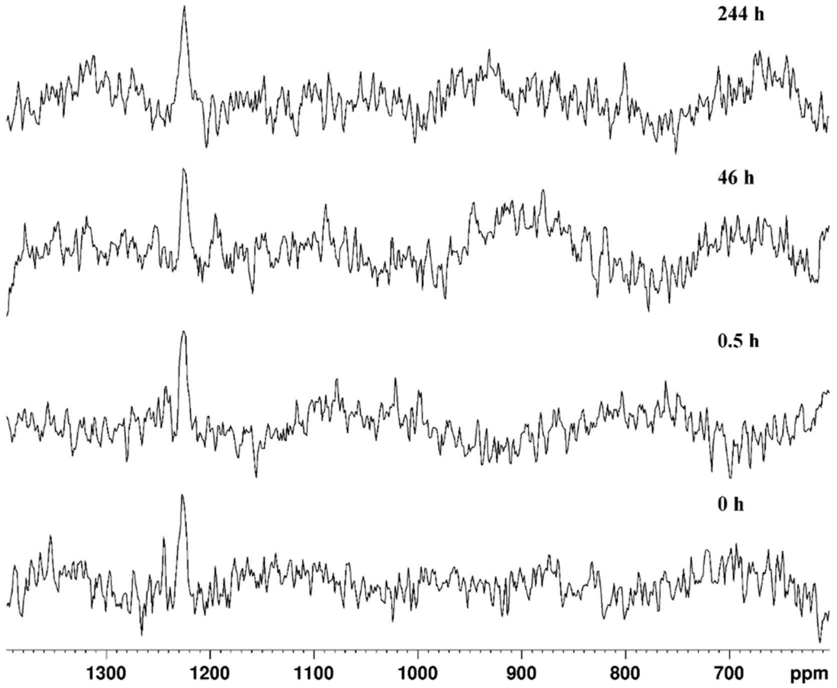


**Figure S26.** $^{195}Pt$ NMR spectra of complex 2 upon addition of glutathione

## Platinum uptake data and LDH activity

**Table S1.** Pt uptake [in ng Pt/ $10^6$ cells] by HL-60 and HT-29 cells after 4 hours of incubation with 10 µM solutions of complexes **1**, **2** and cisplatin (CDDP):

| | **Total Cellular Uptake** | **Cytosolic Fraction** | **Membrane/ Particulate Fraction** | **Nuclear Fraction** | **Cytoskeletal Fraction** |
|---|---|---|---|---|---|
| HT-29_**CDDP** | 19.8 ± 1.0 | 2.9 ± 0.1 | 3.3 ± 0.2 | 0.8 ± 0.01 | 5.8 ± 0.2 |
| HT-29_ **1** | 308.0 ± 15.0 | 155.0 ± 7.6 | 116.0 ± 5.6 | 18.3 ± 0.9 | 12.4 ± 0.5 |
| HT-29_ **2** | 80.9 ± 4.0 | 8.4 ± 0.4 | 33.0 ± 1.4 | 5.7 ± 0.2 | 34.5 ± 1.5 |
| HL-60_**CDDP** | 30.8 ± 1.3 | 5.1 ± 0.3 | 3.8 ± 0.2 | 1.1 ± 0.1 | 16.8 ± 0.7 |
| HL-60_ **1** | 154.0 ± 7.3 | 97.3 ± 4.9 | 33.5 ± 1.4 | 5.9 ± 0.3 | 1.3 ± 0.1 |
| HL-60_ **2** | 255.0 ± 12.5 | 47.9 ± 2.4 | 60.1 ± 2.8 | 6.1 ± 0.3 | 134.0 ± 6.0 |

**Table S2.** Pt uptake in cellular fractions [in pmol Pt/ µg protein] of HL-60 and HT-29 cells after 4 hours of incubation with 10 µM solutions of complexes **1**, **2** and cisplatin (CDDP).

| | **Total Cellular Uptake** | **Cytosolic Fraction** | **Membrane/ Particulate Fraction** | **Nuclear Fraction** | **Cytoskeletal Fraction** |
|---|---|---|---|---|---|
| HT-29_**CDDP** | 0.413 ± 0.009 | 0.060 ± 0.001 | 0.069 ± 0.001 | 0.017 ± 0.0001 | 0.121 ± 0.001 |
| HT-29_ **1** | 6.776 ± 2.296 | 3.410 ± 0.581 | 2.552 ± 0.326 | 0.403 ± 0.008 | 0.273 ± 0.004 |
| HT-29_ **2** | 1.435 ± 0.103 | 0.149 ± 0.001 | 0.585 ± 0.017 | 0.101 ± 0.001 | 0.612 ± 0.019 |
| HL-60_**CDDP** | 1.305 ± 0.085 | 0.216 ± 0.002 | 0.161 ± 0.001 | 0.047 ± 0.0002 | 0.712 ± 0.025 |
| HL-60_ **1** | 10.964 ± 0.610 | 6.927 ± 0.299 | 2.385 ± 0.184 | 0.420 ± 0.009 | 0.093 ± 0.001 |
| HL-60_ **2** | 10.803 ± 0.535 | 2.029 ± 0.206 | 2.546 ± 0.124 | 0.258 ± 0.003 | 5.677 ± 0.611 |

**Table S3.** Total protein content in HL-60 and HT-29 cells after 4 hours of incubation with 10 µM solutions of complexes **1**, **2** and cisplatin (CDDP). Ko stands for untreated control cells.

| **Compounds** | **Total Protein [µg]** | |
|---|---|---|
| | **Total** | **STDEV** |
| HT-29 _ Ko | 260 | 6,8 |
| HT-29 _ CDDP | 246 | 6,7 |
| HT-29 _ Compl. **1** | 233 | 9,6 |
| HT-29 _ Compl. **2** | 289 | 9,5 |
| HL-60 _ Ko | 137 | 3,8 |
| HL-60 _ CDDP | 121 | 1,4 |
| HL-60 _ Compl. **1** | 72 | 2,7 |
| HL-60 _ Compl. **2** | 121 | 1,1 |

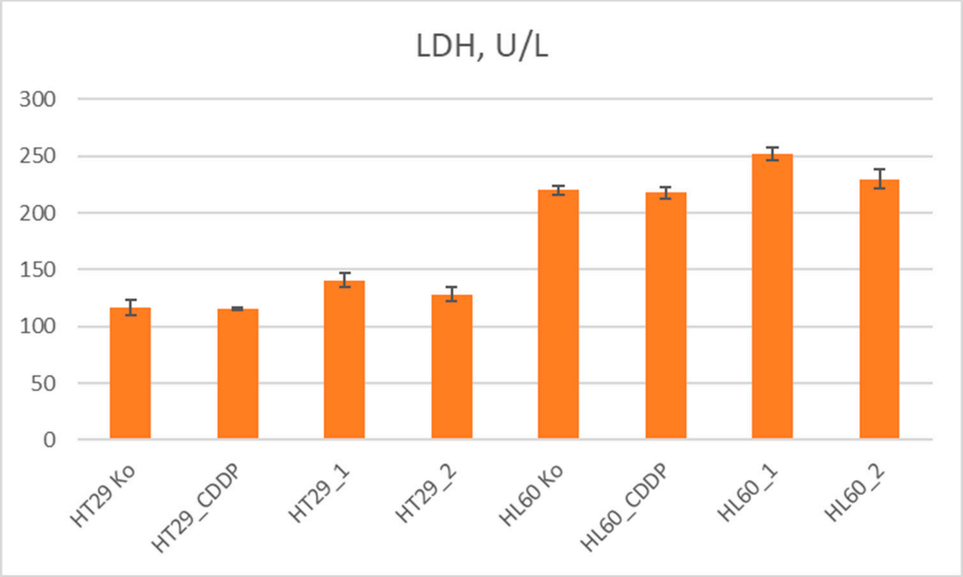


**Figure S27**. LDH activity data in the supernatants of the HL-60 and HT-29 cells after 4 hours of incubation with 10 µM solutions of complexes **1**, **2** and cisplatin (CDDP). Ko stands for untreated control cells.
